# Analysis of Mutations and Dysregulated Pathways Unravels Carcinogenic Effect and Clinical Actionability of Mutational Processes

**DOI:** 10.3389/fcell.2021.768981

**Published:** 2021-11-24

**Authors:** Zedong Jiang, Gaoming Liao, Yiran Yang, Yujia Lan, Liwen Xu, Min Yan, Yao Zhou, Jiali Zhu, Wei Liu, Jing Bai, Yun Xiao, Xia Li

**Affiliations:** ^1^ College of Bioinformatics Science and Technology, Harbin Medical University, Harbin, China; ^2^ Key Laboratory of High Throughput Omics Big Data for Cold Region’s Major Diseases in Heilongjiang Province, Harbin, China

**Keywords:** mutational process, mutational signature, mutation, homologous recombination proficient, APOBEC mutational signature

## Abstract

Somatic mutations accumulate over time in cancer cells as a consequence of mutational processes. However, the role of mutational processes in carcinogenesis remains poorly understood. Here, we infer the causal relationship between mutational processes and somatic mutations in 5,828 samples spanning 34 cancer subtypes. We found most mutational processes cause abundant recurrent mutations in cancer genes, while exceptionally ultraviolet exposure and altered activity of the error-prone polymerase bring a large number of recurrent non-driver mutations. Furthermore, some mutations are specifically induced by a certain mutational process, such as IDH1 p.R132H which is mainly caused by spontaneous deamination of 5-methylcytosine. At the pathway level, clock-like mutational processes extensively trigger mutations to dysregulate cancer signal transduction pathways. In addition, APOBEC mutational process destroys DNA double-strand break repair pathway, and bladder cancer patients with high APOBEC activity, though with homologous recombination proficient, show a significantly longer overall survival with platinum regimens. These findings help to understand how mutational processes act on the genome to promote carcinogenesis, and further, presents novel insights for cancer prevention and treatment, as our results showing, APOBEC mutagenesis and HRD synergistically contributed to the clinical benefits of platinum-based treatment.

## Introduction

Mutational processes, the biological activities that generated mutations, were vital risk factors for carcinogenesis (Pfeifer, 2010; Peña-Diaz et al., 2012). Nik-Zainal et al. (2012), Alexandrov et al. (2013a) developed a mathematical method to describe the mutational processes in an individual cancer genome by using mutational signatures, which greatly improved our understanding of mutational processes. Researchers (Alexandrov et al., 2013b; Alexandrov et al., 2020) have identified a large number of mutational signatures in the cancer genome, some of which are associated with some exogenous or endogenous mutation processes, such as age, exposures to ultraviolet, DNA repair pathways deficiency, and APOBEC activity.

To gain biological insights, researchers portrayed correlation relationships between cancer gene mutations and different mutational processes in a variety of cancer types based on population samples (Degasperi et al., 2020; Poulos et al., 20181007; Temko et al., 2018). But this analysis lacks a causal explanation, that is, it could not distinguish between mutations as a cause or a consequence of the mutational process. Fortunately, the sequence characteristics of mutations lend credence to causal associations. Some mutational processes generate specific patterns of mutations, and this preference will increase the possibility of certain mutations in the genome. For example, POLE p.P286R [C(C>G)T] and PTEN p.R130Q [T(C>T)G] are significantly associated with SBS10 activity in UCEC. The trinucleotide context of C(C>G)T is not frequently observed in SBS10, indicating that the POLE p.P286R mutation is basic in the presence of SBS10 in cancer. This trinucleotide context of T(C>T)G is frequently mutated in SBS10, suggesting that this driver mutation likely arose as a direct result of exposure to the mutagenic processes underlying SBS10.

Some studies have described examples of mutations induced by mutation processes, thus providing the first glimpse of the carcinogenesis of mutational processes. For example, APOBEC mutational processes were found to generate *PIK3CA* helical domain mutations (Henderson et al., 2014) and *FGFR* S249C (Shi et al., 2019) to contribute to tumor development. And, Li et al. found that chemotherapy-induced mutagenesis caused the drug resistance mutations such as *NT5C2* (Li et al., 2020). These results indicate that the mutational process causes specific mutations to perform oncogenic functions. However, the mutation panorama shaped by various mutational processes is not exhaustively understood.

Therefore, we systematically constructed the mutation landscape induced by various mutational processes in 5,828 samples spanning 34 cancer types and subtypes and dissected the carcinogenic ways of various mutational processes. More importantly, through the extensive mutation spectrum of mutational processes, we can understand the carcinogenic risk of mutational processes and pathways they destroy, providing a comprehensive insight into the role of the mutational process in cancer initiation and development. Typically, we found that mutations that accumulated with age were widely enriched in cancer signaling pathways, indicating a key role of aging in cancer development. And APOBEC mutational process destroys DNA double-strand break repair pathway, revealing a potential clinical application value.

## Materials and Methods

### Datasets Used in the Study

We used the mutation annotation format (MAF) file (version 2.8) provided by the MC3 (Multi-Center Mutation Calling in Multiple Cancers) group within the TCGA Network (Ellrott et al., 2018). The mutation data can be found here (https://gdc.cancer.gov/about-data/publications/mc3-2017) denoted as “Mutations - mc3.v0.2.8.PUBLIC.maf.gz”. For somatic mutations FILTER values were required to be one of PASS, wga, or native_wga_mix, and only single base substitutions were retained in this study. In mutational signature refitting, the fitting accuracy will increase with the increase of mutation size (Supplementary Figure S1). Therefore, to ensure the accuracy of identifying the contribution of each mutational process, we excluded samples with fewer than 50 mutations. The samples were annotated with molecular subtypes based on genomic characterization from TCGA Research Network tumor-specific publications (Sanchez-Vega et al., 2018). After removing some cancer types with smaller sample sizes, we finally enrolled 5,828 samples across 34 cancer types and subtypes for downstream analyses (Supplementary Table S1). Cancer genes were defined using a recent study by Bailey et al. (2018).

### Mutational Signature Exposures for Each Sample

We obtained all single base substitution mutational signatures from Alexandrov et al. (2020). Then, an enhanced NNLS framework (Alexandrov et al., 2020) was applied to determine the proportion of mutations attributable to each of the substitution mutational signatures. This framework included the following optimization processes to minimize the signature bleeding effect, and an optimal mutational signature set was finally determined for each sample. In the first step, a reasonable set of mutational signatures was selected for each cancer type based on prior knowledge (Alexandrov et al., 2013b; Alexandrov et al., 2020), the detail was in Supplementary Table S6. Further, for each sample, we filtered mutational signatures based on transcriptional strand bias and the total number of somatic mutations. In the second step, during the NNLS fitting process, the mutational signatures that contribute less to the fitting will be sequentially removed. In the third stage, some of the rest mutational signatures will be added provided that it increases the fitting accuracy.

## Inferring Causal Relationship Between the Mutational Process and Mutations.

The probability that a mutation was caused by a signature was calculated using an approach described previously (Morganella et al., 2016; Li et al., 2020). Let $S_{K}$ represent the mutational signature exposure vector of the signature for a given sample, and c = 1, 2, …, 96 represent each of the 96 possible trinucleotide mutation types. Each of the k signatures mutated each of these 96 trinucleotide mutation types c with a probability $P_{c,\;k}$ (ranging from 0 to 1) where the sum of the probabilities for a given signature across all 96 trinucleotide mutation types is 1. The probability that a mutation of interest m (at trinucleotide mutation type c) was caused by a specific signature i is calculated as:

$$P\left(i|m\right)=\frac{s_{i}\times P_{c,i}}{\sum \left(s_{k}\times P_{c,k}\right)}$$

Then, the probability of signatures was merged according to common etiologies. For example, SBS2 and SBS13 were merged into one APOBEC mutational signature (labeled as SBS2/13), and SBS6, 14, 15, 20, 21, 26 were merged into the MMR related signature (labeled as SBS6*). Finally, for each mutation, the mutational process with the highest probability was selected as its associated mutational process. The association with a probability of less than 0.5 was marked as “Ambiguous” and given no analysis.

### The Risk of Mutational Processes Inducing Cancer Gene Mutations

A robust linear regression (Huber and Lovric, 2011) was used to evaluate linear dependencies between the number of non-silent cancer gene mutations and the number of all mutations affected by a certain mutational signature in each cancer subtype cohort. The P values of the regression model were corrected for multiple hypothesis testing using the Holm-Bonferroni method (Holm, 1979). The line’s slope of robust regression was then defined as the carcinogenic risk caused by the mutational process, which represents the probability that this mutation was a non-silent driver mutation when the mutational process caused a mutation in the exon. This analysis was performed with the rlm function in the “MASS” package (Ripley, 2002).

## Identification of Mutations Caused by a Mutational Process Specifically

We calculated normalized entropy (Tokheim et al., 2016; Bailey et al., 2018) to characterize mutations on their diversity of effects by mutational processes at cancer-type specific levels:

$$E=\frac{-\sum_{i=1}^{n}p\left(i\right)\log_{2}\left(p\left(i\right)\right)}{\log_{2}\left(n\right)}$$

where, for each mutation in a cohort, n is the total number of mutations, and p(i) represents the proportion affected by the i-th mutational process. This score takes values between 0 and 1, where a value closer to 0 indicates that the mutation was dominantly affected by a certain mutational process. Then, for a given mutation and a mutational process, we modeled the mutation number caused by the dominant mutational process as a binomial distribution with N trials with success probability p, and binomial distribution test was used to infer whether the dominant mutational process effect was over-presented by testing the null hypothesis H0: *p* = 0.5 against the alternative hypothesis H1: *p* > 0.5. Then the P values were corrected for multiple hypothesis testing using the Holm-Bonferroni method (Holm, 1979).

### Analysis of Pathways Disturbed by Mutational Processes

For a given mutational process A in a cancer type or subtype, we used the following method to identify related pathways (Supplementary Figure S9). Firstly, we prioritized genes based on the number of non-synonymous mutations caused by A for each cancer type. Since many mutations caused by A have the same frequency, we combined the posterior probabilities of associations between A and mutations to rank the genes. Then, pathway enrichment analysis in ranked lists of candidate genes is carried out with a hypergeometric test described previously (Paczkowska et al., 2020). Biological pathways of the Reactome database (Joshi-Tope et al., 2004) were used as the source of human pathways, where large general gene sets with more than 500 genes and small specific gene sets with less than 10 genes were removed. The ranked hypergeometric P-value was computed for all pathways and resulting P values are corrected for multiple testing using the Holm-Bonferroni method (Holm, 1979). Finally, for each pathway, we integrated the evidence from all cancer types by merging all P values using Brown’s extension (Brown, 1975) of the Fisher’s combined probability test. And significant pathways were reported by *p* < 0.05. EnrichmentMap (Merico et al., 2010) and AutoAnnotate (Kucera et al., 2016) application of Cytoscape (Shannon et al., 2003; Cline et al., 2007) were used for network visualization of similar pathways with stringent pathway similarity scores (Jaccard and overlap combined coefficient 0.6) and their coloring according to cancer types. We manually chose the most representative name for a group of similar pathways and processes based on prior knowledge.

### Evaluating the Clinical Application of the APOBEC Mutational Process

We acquired 88 BLCA patients who underwent platinum-based adjuvant chemotherapy after tissue collection based on the time of sampling and first treatment. First, we explored the clinical net income of two prognostic models based on two factors: HRD score and combination of the contribution of APOBEC mutational process and HRD score. This analysis was performed utilizing the “DCA” package. Then, we evaluated the prognostic power of APOBEC activity. Referred to previous studies (Telli et al., 2016), patients with HDR scores greater than 42 or BRCA1/2 mutation were defined as HR-deficient, and the AOBEC-related mutational processes contribution greater than 0.25 were defined as APOBEC-high. The survival curves were calculated with Kaplan-Meier estimation, and the differences between survival curves were calculated by log-rank test. The hazard ratio, multivariate analysis adjusting for clinical parameters was determined through a Cox proportional hazards model. Survival analysis was carried out using the “survminer” and “survival” R packages.

### Statistics Analysis

All statistical analyses were performed with R statistical software version 3.5.2 (http://www.R-project.org). The significance of differences between the two groups was determined by Wilcoxon rank-sum test. The Chi-square test was used to determine the significance of the overlap between the two groups. We used copy number burden as a mediator to analyze the relationship between mutations caused by APOBEC and HR-deficient using the “mediation” package. A Linear mixed-effect model was used to associate APOBEC-related mutational processes contribution with HRD contribution across cancer types using the “lme4” package.

## Results

### Mutational Processes Exhibit Diverse Carcinogenic Risks in Human Cancers

**FIGURE 1:**
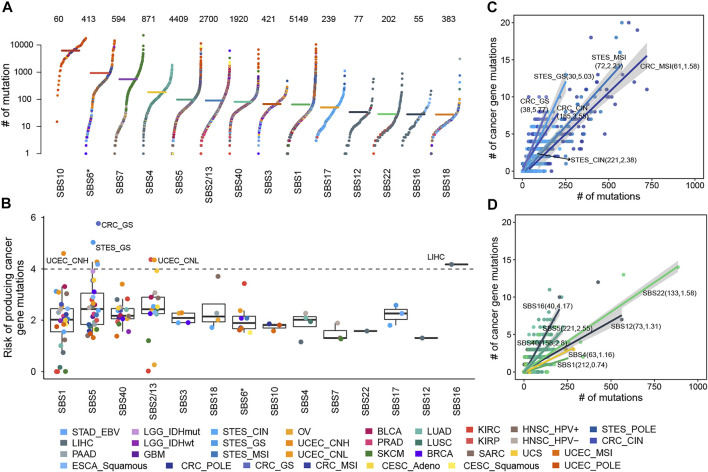
The cancer gene mutation risk of mutational processes. **(A)** The mutation burdens induced by mutational processes. Each dot represents a sample colored by the cancer type while the horizontal lines are the median numbers of mutations in the respective cancer types. The total number of samples affected by each mutational process is marked at the top of the figure. The y axis (log scaled) shows the number of mutations while different mutational signatures are ordered on the x-axis based on their median numbers of mutations. **(B)** The cancer gene mutation risk of each mutational process in each cancer type or subtype was represented as a dot colored according to cancer type or subtype. Boxplots represent the median and interquartile range (IQR). **(C)** The cancer gene mutation risk of SBS5 in GIACs. Each dot represents a sample colored by cancer subtype, and the line corresponding to each cancer subtype represents the linear relationship between the number of cancer gene mutations and total mutations caused by SBS5. **(D)** The cancer gene mutation risk of several mutational processes in LIHC. Each dot represents a sample colored by the mutational process, and each line represents the linear relationship between the number of cancer gene mutations and total mutations caused by the mutational process. In **B** and **C**, the sample size and carcinogenic risk of mutational processes were shown in parentheses separated by commas.

In 5,828 samples, we calculated the contribution of various mutational processes which can leave mutations in the genome, increasing the risk of carcinogenesis. Then the causal relationships between mutational processes and mutations were identified (details see Methods), to estimate the carcinogenic risk of mutational processes by calculating their mutagenic ability and risk of causing non-silent mutations affecting cancer genes. As expected, the mutational processes mediated by DNA damage repair, including the altered activity of the error-prone polymerase (SBS10) and defective DNA mismatch repair (MMR defects, SBS6*), generate huge numbers of mutations in samples (median mutation load: 6,227 and 927; Figure 1A), which may reflect a strong carcinogenic ability.

Further, we found the number of non-silent mutations in driver genes and the total number of exon mutations caused by the mutational process were highly correlated and displayed a linear relationship, albeit with different ratios across cancer types (Supplementary Figure S2), suggesting the different but constant carcinogenic risks induced by mutational processes. Thus, we leveraged robust linear regression to evaluate these linear dependencies (details see Methods). Our results showed that fourteen mutational signatures showed a stable carcinogenic risk in at least one cancer type (adjusted P < 0.05 ; Figure 1B and Supplementary Table S3). For example, we calculated that 1.28 out of 100 exon mutations (1.28%) contributed by UV exposure in melanoma (SKCM) were expected to affect known cancer genes (adjusted $P\;<\;2.2\times 10^{-16}$ , $R^{2}\;=\;0.93$ ).

The risk values of cancer gene mutations across cancer types contributed by a mutational process showed substantial variation, especially SBS1, SBS5, and APOBEC-mediated mutational processes (Figure 1B, Supplementary Figure S3). SBS1 and SBS5 were found to correlate with age at diagnosis, showing clock-like properties (Alexandrov et al., 2020; Alexandrov et al., 2015). Typically, SBS5 induced different cancer gene mutation risk across gastrointestinal adenocarcinomas (GIACs), being high in genomically stable gastroesophageal cancer (STES GS, 5.03%, adjusted $P\;=\;2.6\times 10^{-9}$ , $R^{2}\;=\;0.50$ ) and colorectal cancer (CRC GS, 5.77%, adjusted $P\;=\;4.1\times 10^{-6}$ , $R^{2}\;=\;0.73$ ) in comparison to GIACs with chromosomal instability and microsatellite instability (Figure 1C, Supplementary Table S3). Further analysis showed that cancer gene mutation risk induced by SBS5 was negatively correlated with mutation load and copy number load (Supplementary Figures S4A,B). However, genomically stable GIACs have low mutation load and copy number load (Supplementary Figures S4C,D). One possible explanation is that genomically unstable tumors have higher DNA damage pressure and lower selection intensity, thus leading to a lower mutation risk in cancer genes. In addition, different mutational processes had various degrees of contributions to cancer gene mutations in a cancer type (Supplementary Figure S3). In liver cancer, SBS16, related to alcohol consumption with strong evidence (Wei et al., 2020), had a high cancer gene mutation risk (4.17%, adjusted $P\;=\;2.7\times 10^{-14}$ , $R^{2}\;=\;0.56$ ), while other processes such as SBS1 had a relatively small contribution to cancer gene mutation (Figure 1D, Supplementary Table S3). This suggested that the toxic effects of alcohol on the liver genome may be more serious than the cumulative effects of clock-like mutational processes.

### The Landscape of High-Frequency Mutations Shaped by Mutational Processes

**FIGURE 2:**
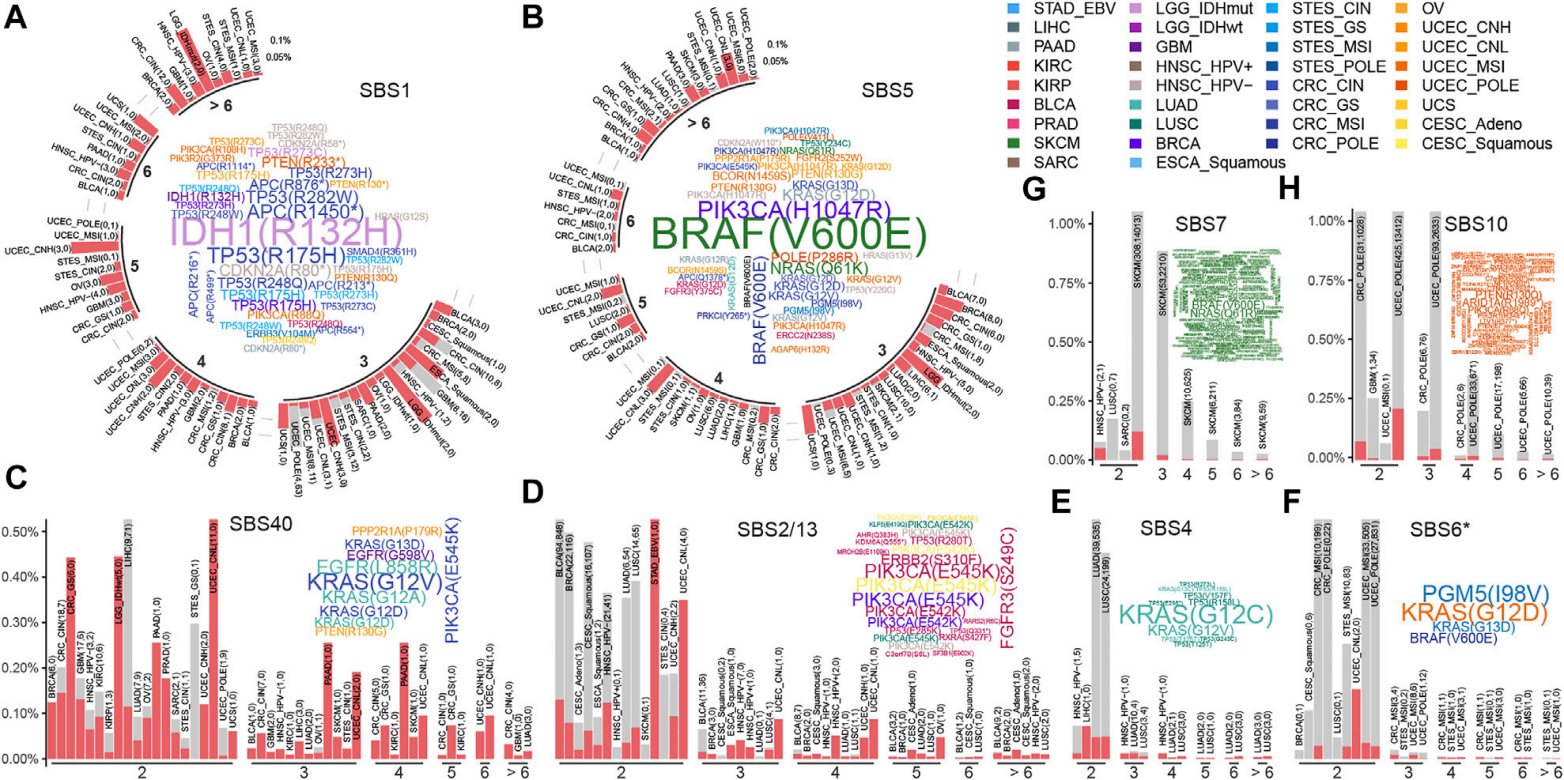
The recurrent mutations shaped by mutational processes. Barplots and word clouds illustrate the recurrent mutation landscapes for **(A)** SBS1, **(B)** SBS5, **(C)** SBS40, **(D)** SBS2/13, **(E)** SBS4, **(F)** SBS6*, **(G)** SBS7, and **(H)** SBS10. Barplot depicts a recurrence pattern, where mutations were binned by their reoccurrence frequency and the height of the bar represented the fraction of mutations in each cancer type or subtype. The numbers of mutations in and out of cancer genes were filled by red and gray respectively and also showed in parentheses separated by commas in each cancer type or subtype. The bar plot is displayed in a circular layout in **(A)** and **(B)**. Word clouds show high-frequency mutations occurring at least 6 times in a cancer type or subtype, of which word size is proportional to the number of mutations and word colored by cancer type or subtype.

When the mutational process inducing mutations, some of them might increase the growth advantage of cancer and would be retained by the selection, showing a trend of high frequency in the sample population. Here, we systematically portrayed the landscape of high-frequency mutations that were shaped by mutational processes. As a result, for two clock-like mutational processes, SBS1 gave rise to 4,782 recurrent mutations across 30 cancer types and subtypes (Figure 2A), and SBS5 resulted in 596 recurrent mutations in 28 cancer types and subtypes (Figure 2B). Especially, there were both 25 mutations induced by SBS1 and SBS5 respectively occurring frequently in samples (at least six samples) across a variety of cancer types. For example, SBS1-mediated mutation p.R132H of *IDH* was observed in 12 glioblastomas (GBM) and 52 low-grade gliomas (LGG) samples. In colorectal cancer, SBS1 mainly caused mutations in *TP53* (such as p.R175H and p.R282W) and *APC* (such as p.R1450* and p.R216*) to act carcinogenic role (Figure 2A). The most frequent mutation induced by SBS5, *BRAF* p.V600E, was found in 93 SKCM samples and 28 MSI CRC samples (Figure 2B). Surprisingly, most of the high-frequency recurrent mutations induced by SBS5 were also induced by SBS40 (Figure 2BC), suggesting the convergence of carcinogenic effects of different mutation processes.

The APOBEC mutational process mainly resulted in mutations in the *PIK3CA*, such as p.E545K and p.E542K in CESC, BLCA, and BRCA (Figure 2D). SBS4, related to tobacco exposure, showed different mutagenicity patterns in lung adenocarcinoma (LUAD) and lung squamous cell carcinoma (LUSC) (Figure 2E). For example, SBS4-mediated *KRAS* mutations, including p.G12C and p.G12V being dominant in many LUAD samples. While a large number of high-frequency mutations (such as p.R158L, p.V157F, and p.R373L) were found in *TP53* across LUSC samples (Figure 2E). MMR defect-associated mutation processes resulted in fewer high-frequency sites, mainly in UCEC and GI samples resulting in *KRAS* (G12/13D) and *BRAF* (V600E) mutations (Figure 2F).

For SBS7 and SBS10, unlike other mutational processes, with were showed high mutagenic ability (Figure 1A), and caused a large number of recurrent non-driving mutations in melanoma and uterine corpus endometrial carcinoma (UCEC), respectively (Figures 2G,H), suggesting that their mutagenicity was stronger than selection effect. In terms of cancer genes, SBS7 caused a large number of *BRAF* and *NRAS* mutations in SKCM, and SBS10 caused some high-frequency mutations in *PTEN* genes in UCEC. Other cancer-specific mutational processes were found to cause rare high-frequency mutation sites (Supplementary Figure S5). This may be due to the small sample size (Supplementary Figure S6) and mutations (Figure 1A) affected by these mutational processes.

### Identifying Mutations Specifically Induced by a Certain Mutational Process

**FIGURE 3:**
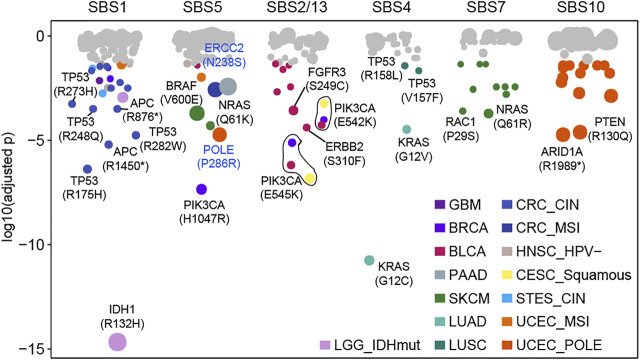
Some mutations caused by a certain mutational process specifically. Each dot represents a specific relationship between mutation and mutational process. P values were obtained from the binomial test after multiple test corrections. Significant dots (adjusted *p* < 0.05) are filled by cancer type and the size of dots indicates the frequency of mutation in the corresponding cancer type or subtype.

Our above results indicated that the mutational processes induced high-frequency cancer gene mutations (Supplementary Figure S7), which was essential for understanding the carcinogenic mechanisms of the mutational processes. Then, we used a binomial distribution test to screen for mutations specifically caused by a certain mutational process (details see Methods). In total, 39 significant specific associations were found among six mutational processes across 15 cancer types (adjusted P < 0.05 ; Figure 3, Supplementary Figure. S8, Supplementary Table S4). For example, 23 associations of SBS1-specific in seven cancer types, including the mutations in *TP53* (p.R175H, p.R248Q, p.R273H, and p.R282W), and the truncated mutations of *APC* and *CDKN2A* in CRC CIN and HPV- HNSC respectively (Figure 3). Indeed, codons of these positions contained CpG dinucleotides, which were susceptible to produce C > T mutation by SBS1 (Alexandrov et al., 2013b). There were nine associations caused by SBS5 specifically, including *PIK3CA* H1047R in HNSC and BRCA, *BRAF* V600E in SKCM and CRC, and *NRAS* Q61K in SKCM (Figure 3). Our results also suggest that SBS5 may have an impact on NER-related mutational phenotype by inducing *ERCC2* p.N238S and *POLE* p.P286R, which are responsible for proofreading and faithful replication of DNA (Rayner et al., 2016).

APOBEC mutagenesis has mutational specificity in TCW motifs (where W corresponds to either A or T) (Burns et al., 2013; Roberts et al., 2013). And 13 APOBEC-related associations were observed in BLCA (*n* = 9), BRCA (*n* = 2), and CESC (*n* = 2), of which p.E542K and p.E545K in *PIK3CA* accounted for 46.2% (6/13) associations across three cancer types (Figure 3). For known cancer-related mutational processes, we found SBS4 specifically included *KRAS* mutations (G12V, G12C) and *TP53* mutations (V457F, R158L) in LUAD and LUSC respectively (Figure 3). Additionally, SBS7 had 10 associations in SKCM (such as *NRAS* Q61R, *RAC1* P29S) and SBS10 had 14 associations in UCEC (such as *PTEN* R130Q, *ARID1A* R1989*) (Figure 3, Supplementary Figure S8). These results suggest that the mutational processes can uniquely affect genomic mutations, leading to specific oncogenic effects.

### The Landscape of Biological Pathways Disrupted by Mutational Processes

**FIGURE 4:**
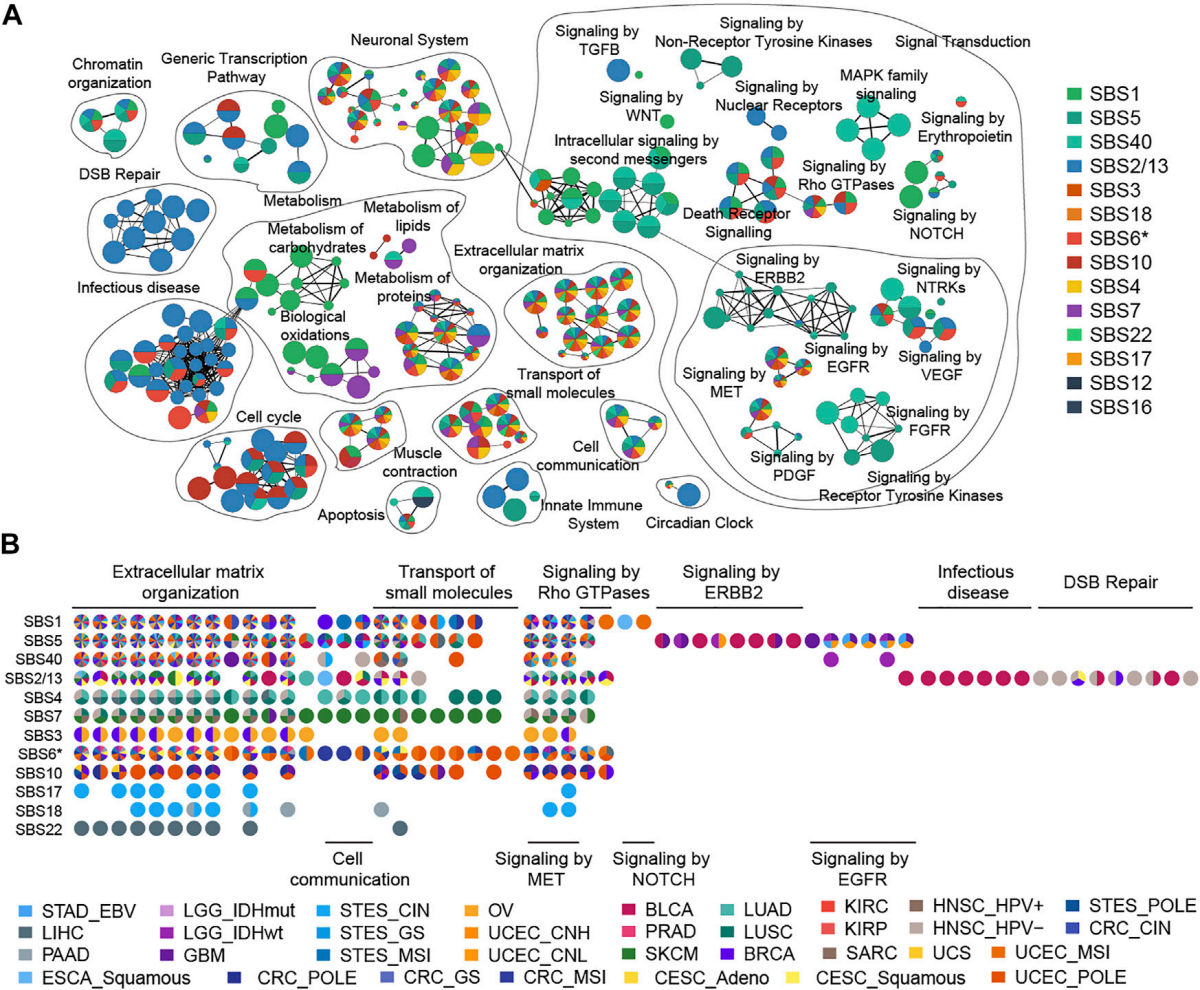
Enrichment map of pathways affected by mutational processes. **(A)** Nodes in the network represent pathways and are colored by associated mutational processes. The node size indicates the number of genes in a pathway. Similar pathways with many common genes are connected and named according to prior knowledge. **(B)** Cancer type evidence of various mutational processes affecting pathways. Nodes in the matrix are filled by cancer-type evidence.

In this study, we used a ranked hypergeometric test to explore the effects of mutational processes on pathways (Supplementary Figure S9, details see Methods). Totally, 14 mutational processes resulted in 294 significantly enriched Reactome pathways (adjusted P < 0.05 , Figure 4, Supplementary Table S5), of which 78 (26.5%) pathways were affected by at least four mutational processes (Supplementary Figure S10A). The major biological themes with these pathways included extracellular matrix organization, cell communication, transport of small molecules, protein metabolism, signal transduction pathways such as MET, rho GTPase, and others that are increasingly recognized in cancer biology (Figure 4) (Trusolino et al., 2010; Walker et al., 2018). In contrast to these associations, a large group of pathways (136, 46.3%) were affected by one mutational process solely, and APOBEC mutagenesis and clock-like mutational processes contributed mostly (Supplementary Figure S10B).

A striking observation was that clock-like mutational processes had a predominant impact on signal transduction pathways evidenced by multiple cancer types (Figure 4A). For example, the influence of SBS1 on the pathway is mainly focused on the NOTCH signal pathway (such as “Signaling by NOTCH1”, $P\;=\;6.6\times 10^{-3}$ ) and metabolic processes associated pathway, such as “Metabolism of carbohydrates” ( $P\;=\;1.4\times 10^{-6}$ ) and “Integration of energy metabolism” ( $P\;=\;1.4\times 10^{-9}$ ) (Figure 4B; Supplementary Figure S11A). SBS5 mainly caused mutations in the ERBB2 (such as “Signaling by ERBB2 in Cancer”, $P\;=\;1.6\times 10^{-4}$ ), EGFR (such as “Signaling by EGFR in Cancer”, $P\;=\;7.5\times 10^{-3}$ ), FGFR (such as “Signaling by FGFR in disease”, $P\;=\;9.1\times 10^{-3}$ ) and Non-RTK (such as “Signaling by PTK6”, $P\;=1.5\times 10^{-3}$ ) signal pathways in various cancer types (Figure 4B; Supplementary Figure S11B). SBS40 presented similar pathway disturbance with SBS5, but additionally affected the MAPK signal pathway (such as “MAPK family signaling cascades”, ) (Supplementary Figure S11C). These findings suggest that aging plays an important role in activating cancer signal transduction pathways.

### The Clinical Actionability of APOBEC Mutational Process Inducing HRD

Previous studies reported that the APOBEC family of proteins plays an important role in the innate immune response against virus infections (Malim, 2009; Stavrou and Ross, 2015; Vieira and Soares, 2013). Indeed, our result revealed that APOBEC mutagenesis induced abnormalities in the virus infection pathway such as “Interactions of Rev with host cellular proteins” ( $P\;=\;1.8\times 10^{-3}$ ) and “Transport of Ribonucleoproteins into the Host Nucleus” ( $P\;=\;6.6\times 10^{-5}$ ) in multiple cancer types (Figure 4B; Supplementary Figure S12A). Additionally, the APOBEC mutational process affected cell cycle (such as “Mitotic Prometaphase”, $P\;=\;1.2\times 10^{-10}$ ) and DSB repair (such as “HDR through Homologous Recombination”, $P\;=\;6.6\times 10^{-4}$ ) pathways in BLCA, BRCA, HNSC, and CESC (Figure 4B; Supplementary Figure S12A), which were supported by evidence-based on gene expression (Kim et al., 2020).

**FIGURE 5:**
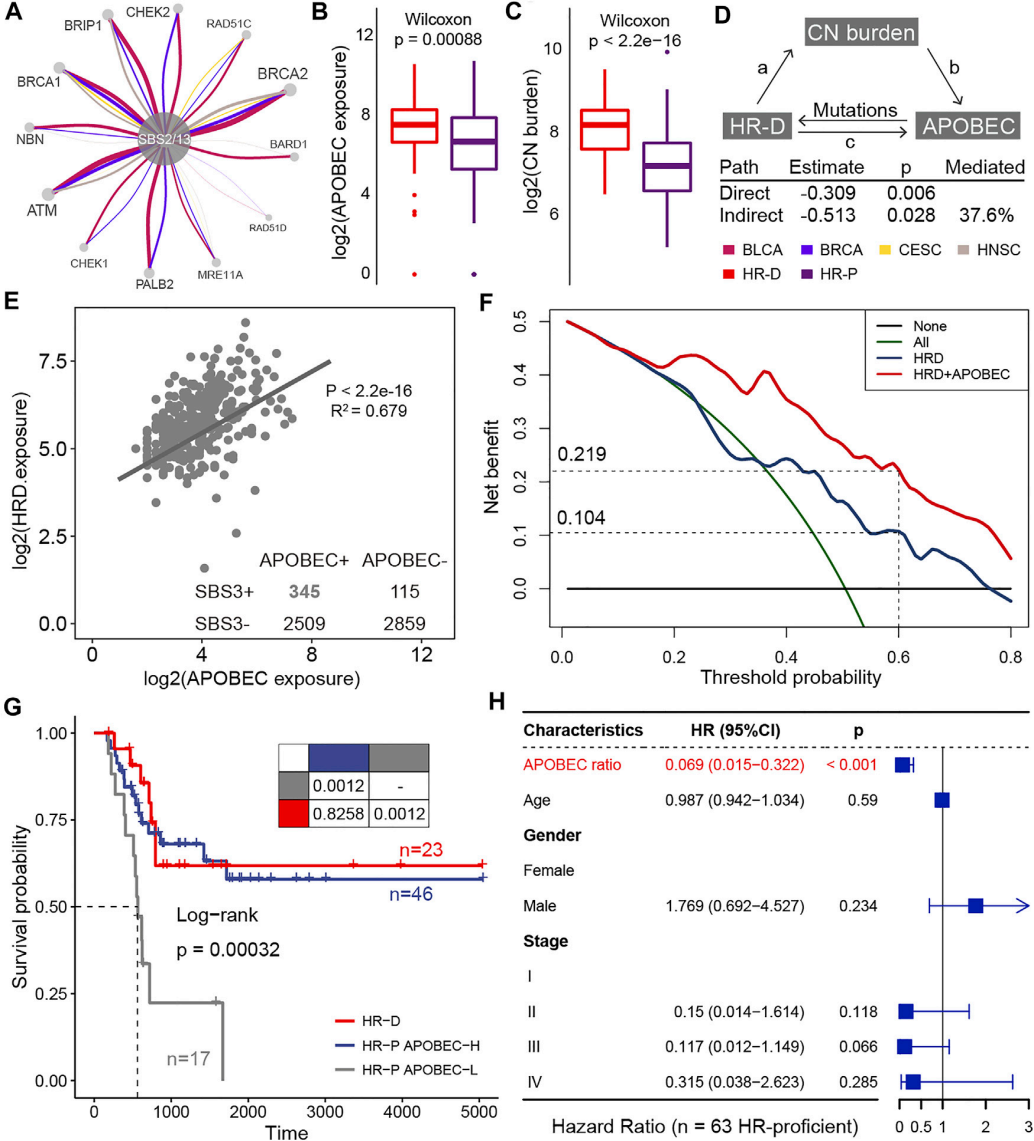
The clinical actionability of APOBEC mutational process. **(A)** HR-related genes affected by APOBEC mutation processes in BLCA, BRCA, CESC, and HNSC. The thickness of the edge connecting two nodes is proportional to the number of associations in all samples. **(B)** Differences in APOBEC exposure between HRD and HRP groups. **(C)** Differences in copy number burden between HRD and HRP groups. **(D)** Mediation analyses examining copy number burden between HRD and APOBEC exposure. **(E)** A scatter plot shows correlations between the log exposure value of APOBEC mutational signatures (SBS2 plus SBS13, x-axes) and HRD signature (SBS3, y-axes). Each dot represents a sample and the line shows best estimates for the slope estimated by mixed effect model in samples that APOBEC mutational signatures and HRD signature co-occurred. **(F)** The decision curve of the net benefit of the two models (HRD score and the combination of HRD score and APOBEC mutational process contribution) for the 5-years survival rate. **(G)** Kaplan–Meier curves for homologous recombination deficient (HR-D; *n* = 23), homologous recombination proficient but APOBEC activity high (HR-P APOBEC-H; *n* = 46), and homologous recombination proficient but APOBEC activity low (HR-P APOBEC-L; *n* = 17) patients who treated with platinum-based adjuvant therapy. Pairwise comparisons of survival curves are presented in the table. **(H)** Hazard ratios for the variables in the Cox regression model. The horizontal bars show the 95% CI; the P values are calculated with two-sided likelihood-ratio tests.

According to this result, we next explored the contribution of APOBEC mutagenesis to homologous recombination repair deficiency (HRD). The APOBEC mutational process caused mutations in HR-related genes, such as BRCA1, BRCA2, and ATM, in large numbers of BLCA, BRCA, CESC, and HNSC samples (Figure 5A; Supplementary Figure S13). We also found that HR-deficient was associated with higher levels of APOBEC exposure in BLCA (Figure 5B, Supplementary Figure S14A). As expected, tumors with deficient HR function provide a huge copy number burden (Figure 5C; Supplementary Figure S14B). We wondered to know whether copy number load can increase the formation of APOBEC-mutagenesis-prone single-stranded DNA (ssDNA). This ssDNA is formed during the 5′→3′ resection that occurs at DNA double-strand breaks during the homology-directed repair (Roberts and Gordenin, 2014). By the mediation analyses, we found copy number burden significantly mediated the association of HR status with APOBEC mutational process exposure (HR = −0.309, *p* = 0.006; Figure 5D). An estimated 37.6% of the association was mediated through copy number burden in BLCA. In addition, APOBEC mutational process and HRD significantly co-occurred in samples ( $P\;<\;2.2\times 10^{-16}$ , $\chi^{2}$ test; Figure 5E). We further used a linear mixed effect model to assess the relationship between the APOBEC mutational process and HRD and found a significantly positive interaction ( $P\;<\;2.2\times 10^{-16}$ , $R^{2}\;=\;0.679$ ; Figure 5E).

HRD is a promising target for platinum-based therapies and poly-ADP ribose polymerase (PARP) inhibitors treatment (Kaufman et al., 2015; Telli et al., 2016). In this study, we asked about the clinical actionability of the positive interaction between the APOBEC mutational process and HRD. We hypothesized that patients with higher APOBEC activity may induce HRD and thus respond to these drugs. To confirm this hypothesis, we curated TCGA BLCA samples who received platinum-based adjuvant therapy and evaluated the impact of APOBEC activity on outcomes. By decision curve analysis, we found considering APOBEC activity resulted in a higher net benefit for platinum-treated patients over a long threshold probability range (Figure 5B). When selecting a threshold probability of 0.6, considering APOBEC activity would benefit an additional 11.5% of patients compared to considering HRD alone (0.219 vs 0.104; Figure 5B). And the overall survival for HR-proficient and APOBEC-high patients (*n* = 46) was comparable to that of patients with HR-deficient (*n* = 23) ( P = 0.826 , log-rank test; Figure 5C), and both two groups had significantly longer overall survival than those patients with HR-proficient and APOBEC-low (*n* = 17) ( P = 0.001 , log-rank test; Figure 5C). We did not find similar results in BRCA, CESC, and HNSC (Supplementary Figure S15). Furthermore, in HR-proficient population, the risk of death is 93.1% lower for APOBEC-high patients than the APOBEC-low cases (hazard ratio: 0.069, 95% confidence interval (CI): 0.015–0.322, P < 0.001 , Cox regression; Figure 5D). From these results, we expect a subset of HR-proficient patients with APOBEC-high would benefit from platinum-based therapy.

## Discussion

**FIGURE 6:**
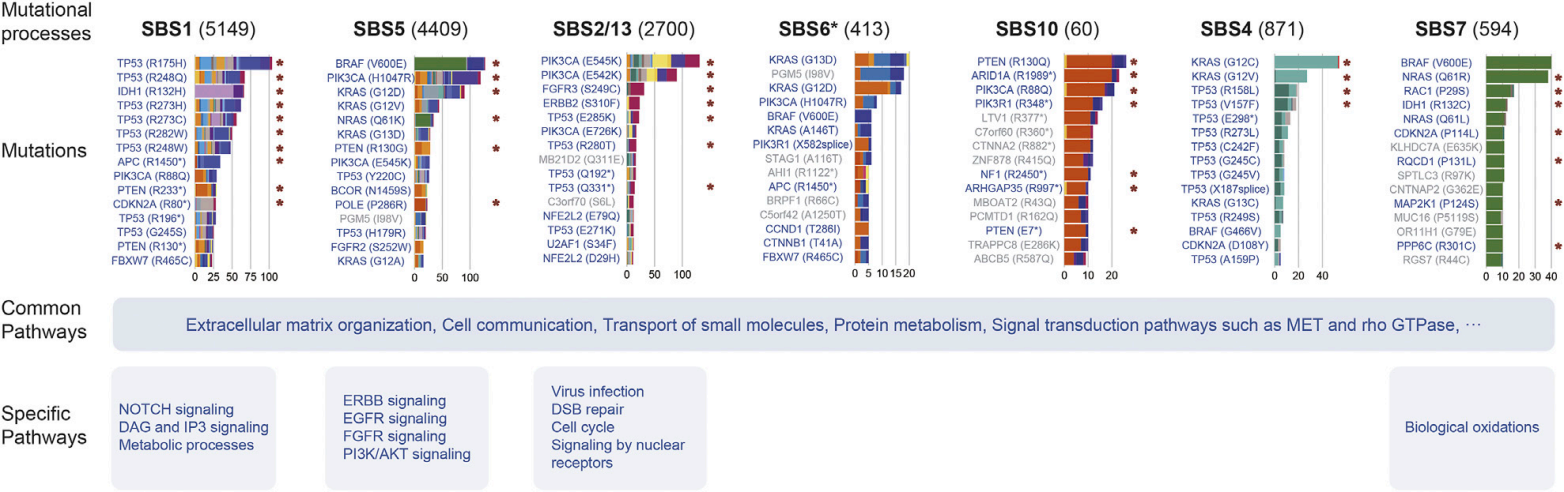
Summary of the effect of mutational processes. This picture exhibits the top 15 mutations and pathways affected by several mutational processes. Barplot shows the mutation number induced by the mutational process, and the filled color of the bar indicates the cancer type or subtype. The red asterisk indicates the specific relationship identified by Results in at least one cancer type. Mutations marked blue are mutations in cancer genes and gray are passenger mutations.

In this study, we described the causal relationship between the mutational process and the genomic mutations (Morganella et al., 2016), and integrated large-scale samples to construct the landscape of mutations and dysregulate biological pathways shaped by mutational processes across multiple cancer types (Figure 6). The genomic landscape is shaped by a balance between the levels of mutations and selective pressures (Temko et al., 2018; Persi et al., 2021). Through observing the highly recurrent mutations caused by each mutational process, we provide evidence for the relative contribution of mutation and selection. Especially, ultraviolet exposure and altered activity of the error-prone polymerase contributed to a large number of recurrent non-driver mutations in melanoma and endometrial cancer, respectively, indicating that the mutation effect of these mutational processes is stronger than selection.

SBS1 and SBS5 were associated with age at diagnosis and persisted throughout the patient’s life, called clock-like mutational signatures (Alexandrov et al., 2015). These two mutational processes inevitably occur frequently in tumor and normal samples, leading to mutations accumulating through a person’s lifetime in different ways (Martincorena and Campbell, 2015; Kim et al., 2020). We found these two mutational processes play key roles in carcinogenesis, showing a link between aging and cancer development. For example, SBS5 brings high carcinogenic risk in stable gastrointestinal tumors. And, though having different mutation context tendencies, they convergently resulted in a large number of mutations in key cancer genes, and extensively activated signal transduction pathways, promoting tumor initiation and progression.

Exogenous mutational processes often cause a large number of genomic damages, involving key cancer genes, playing a crucial role in carcinogenesis. We found UV exposure induced numerous *BRAF* and *NRAS* mutations in SKCM, and the smoking-related mutational process caused *KRAS* and *TP53* mutations in LUAD and LUSC samples respectively. Also, carcinogenic risk analysis indicates that alcohol exposure and aristolochic acid exposure bring a higher risk of cancer gene mutation than the age-accumulated way in liver cancer. Thus, it is necessary to avoid exogenous exposures through human behavior and government control policies.

Damaged DNA repair (DDR) function can cause genomic instability, which is a typical feature of cancer hallmark (Negrini et al., 2010). Our results highlight that some endogenous mutational processes lead to DNA damage repair deficiency. Mutations in NER-related genes, *ERCC2* and *POLE*, were mainly induced by SBS5, indicating that SBS5 plays an important role in blocking NER. In addition, the APOBEC mutational process causes mutations in the DSB pathway, inducing an HRD phenotype. Especially, in BLCA, the deficient HR function was able to increase APOBEC mutation levels through genomic instability, demonstrating a synergistic effect between APOBEC and HRD. These results suggest that these mutational processes not only possess their mutagenic capacity but also activate DNA repair damage-related mutational processes potentially. These mutational processes may leave a large number of mutations in the genome within a short time by this way, of which advantageous mutations may promote tumor evolution, metastasis, and resistance to chemotherapy drugs.

Patients with defects in DNA repair mechanisms can benefit from synthetically lethal therapeutic interventions (Van Allen et al., 2014; Mateo et al., 2015) and immunotherapy (Wang et al., 2019), which may provide a unique clinical application of mutational processes. Sequencing technology provides a cross-sectional snapshot of a patient’s genome, and although we do not find HRD phenotype at this moment, this patient could still benefit from DDR-based therapies as highly APOBEC activity may induce HRD during cancer progression. Indeed, bladder cancer patients with high APOBEC activity, even though HR-proficient, show a significantly longer overall survival with platinum regimens, providing evidence for this idea. This will help to reconcile the paradox that patients with low HRD mutation signature exceptionally respond to the platinum-based drug, and highlight the potential value of considering APOBEC activity in platinum-based therapy. This case raises a meaningful topic that APOBEC activity may help refine decisions on using synthetically lethal therapy, and needs to be validated in more data sets and prospective studies.

In terms of the method that determines the causal association between mutational signature and mutation, it relies on the trinucleotide contexts favored by the mutational process. The similarity of the mutational processes will present a challenge for the accuracy of signature assignments to each mutation. For instance, the APOBEC enzyme mainly causes C > T mutations in the CpG site, so it may be relatively easy to assign a particular mutation to an APOBEC mutational process (SBS2/13). However, some mutational processes, such as SBS5, are rather trickier due to lacking distinctive mutational trinucleotide peaks, exhibiting a flat distribution. As a result, in a sample, the probability of a mutation affected by two mutational processes may be similar and low, leading to the ambiguous assignment. In recognition of this, therefore, we adopt the maximum likelihood with probability threshold approach (Morganella et al., 2016) to achieve our analysis. This process could remove many vaguest assignments to properly mitigate this impact.

In summary, our results presented a comprehensive landscape of the effects of mutational processes on the genome, which was necessary for us to explicitly understand the role of mutational processes in carcinogenesis. And our results provide an extra clinical actionability of mutational processes from an evolutionary perspective.

## Data Availability

All data used in this study were downloaded from public repositories. The TCGA mutation data was downloaded from https://gdc.cancer.gov/about-data/publications/mc3-2017 denoted as “Mutations—mc3.v0.2.8.PUBLIC.maf.gz” (Ellrott et al., 2018). Mutational signatures were downloaded from https://www.synapse.org/#!Synapse:syn12026190.

## References

[B1] Alexandrov L. B. Nik-Zainal S. Wedge D. C. Aparicio S. A. Behjati S. Biankin A. V. (2013). Signatures of Mutational Processes in Human Cancer. Nature 500 (7463), 415–421. Epub 2013/08/16PubMed PMID: 23945592; PubMed Central PMCID: PMCPMC3776390. 10.1038/nature12477 23945592 PMC3776390

[B2] Alexandrov L. B. Jones P. H. Wedge D. C. Sale J. E. Campbell P. J. Nik-Zainal S. (2015). Clock-like Mutational Processes in Human Somatic Cells. Nat. Genet. 47 (12), 1402–1407. Epub 2015/11/10PubMed PMID: 26551669; PubMed Central PMCID: PMCPMC4783858. 10.1038/ng.3441 26551669 PMC4783858

[B3] Alexandrov L. B. Kim J. Kim J. Haradhvala N. J. Huang M. N. Tian Ng A. W. (2020). The Repertoire of Mutational Signatures in Human Cancer. Nature 578 (7793), 94–101. Epub 2020/02/07PubMed PMID: 32025018; PubMed Central PMCID: PMCPMC7054213. 10.1038/s41586-020-1943-3 32025018 PMC7054213

[B4] Alexandrov L. B. Nik-Zainal S. Wedge D. C. Campbell P. J. Stratton M. R. (2013). Deciphering Signatures of Mutational Processes Operative in Human Cancer. Cel Rep. 3 (1), 246–259. Epub 2013/01/16PubMed PMID: 23318258; PubMed Central PMCID: PMCPMC3588146. 10.1016/j.celrep.2012.12.008 PMC3588146 23318258

[B5] Bailey M. H. Tokheim C. Porta-Pardo E. Sengupta S. Bertrand D. Weerasinghe A. (2018). Comprehensive Characterization of Cancer Driver Genes and Mutations. Cell 173 (2), 371–e18. e18Epub 2018/04/07PubMed PMID: 29625053; PubMed Central PMCID: PMCPMC6029450. 10.1016/j.cell.2018.02.060 29625053 PMC6029450

[B6] Brown M. B. (4001). A Method for Combining Non-independent, One-Sided Tests of Significance. Biometrics 31 (4), 987–992.

[B7] Burns M. B. Lackey L. Carpenter M. A. Rathore A. Land A. M. Leonard B. (2013). APOBEC3B Is an Enzymatic Source of Mutation in Breast Cancer. Nature 494 (7437), 366–370. Epub 2013/02/08PubMed PMID: 23389445; PubMed Central PMCID: PMCPMC3907282. 10.1038/nature11881 23389445 PMC3907282

[B8] Cline M. S. Smoot M. Cerami E. Kuchinsky A. Landys N. Workman C. (2007). Integration of Biological Networks and Gene Expression Data Using Cytoscape. Nat. Protoc. 2 (10), 2366–2382. Epub 2007/10/20PubMed PMID: 17947979; PubMed Central PMCID: PMCPMC3685583. 10.1038/nprot.2007.324 17947979 PMC3685583

[B9] Degasperi A. Amarante T. D. Czarnecki J. Shooter S. Zou X. Glodzik D. (2020). A Practical Framework and Online Tool for Mutational Signature Analyses Show Intertissue Variation and Driver Dependencies. Nat. Cancer 1 (2), 249–263. Epub 2020/03/03PubMed PMID: 32118208; PubMed Central PMCID: PMCPMC7048622. 10.1038/s43018-020-0027-5 32118208 PMC7048622

[B10] Ellrott K. Bailey M. H. Saksena G. Covington K. R. Kandoth C. Stewart C. (2018). Scalable Open Science Approach for Mutation Calling of Tumor Exomes Using Multiple Genomic Pipelines. Cell Syst 6 (3), 271–e7. e7PubMed PMID: 29596782; PubMed Central PMCID: PMCPMC6075717. Epub 2018/03/30. 10.1016/j.cels.2018.03.002 29596782 PMC6075717

[B11] Henderson S. Chakravarthy A. Su X. Boshoff C. Fenton T. R. (2014). APOBEC-mediated Cytosine Deamination Links PIK3CA Helical Domain Mutations to Human Papillomavirus-Driven Tumor Development. Cel Rep. 7 (6), 1833–1841. Epub 2014/06/10PubMed PMID: 24910434. 10.1016/j.celrep.2014.05.012 24910434

[B12] Holm S. (1979). A Simple Sequentially Rejective Multiple Test Procedure. Scand. J. Stat. 6 (2), 65–70.

[B13] Huber P. J. (2011). “Robust Statistics,” in International Encyclopedia of Statistical Science. Editor Lovric M (Berlin, Heidelberg: Springer Berlin Heidelberg), 1248–1251. 10.1007/978-3-642-04898-2_594

[B14] Joshi-Tope G. Gillespie M. Vastrik I. D'Eustachio P. Schmidt E. de Bono B. (2004). Reactome: a Knowledgebase of Biological Pathways. Nucleic Acids Res. 33 (Database issue), D428–D432. Epub 2004/12/21PubMed PMID: 15608231; PubMed Central PMCID: PMCPMC540026. 10.1093/nar/gki072 PMC540026 15608231

[B15] Kaufman B. Shapira-Frommer R. Schmutzler R. K. Audeh M. W. Friedlander M. Balmaña J. (2015). Olaparib Monotherapy in Patients with Advanced Cancer and a Germline BRCA1/2 Mutation. Jco 33 (3), 244–250. Epub 2014/11/05PubMed PMID: 25366685; PubMed Central PMCID: PMCPMC6057749. 10.1200/JCO.2014.56.2728 PMC6057749 25366685

[B16] Kim Y.-A. Wojtowicz D. Sarto Basso R. Sason I. Robinson W. Hochbaum D. S. (2020). Network-based Approaches Elucidate Differences within APOBEC and Clock-like Signatures in Breast Cancer. Genome Med. 12 (1), 52. Epub 2020/05/31PubMed PMID: 32471470; PubMed Central PMCID: PMCPMC7260830. 10.1186/s13073-020-00745-2 32471470 PMC7260830

[B17] Kucera M. Isserlin R. Arkhangorodsky A. Bader G. D. (2016). AutoAnnotate: A Cytoscape App for Summarizing Networks with Semantic Annotations. F1000Res, 5, F1000Res1717. Epub 2016/11/11PubMed PMID: 27830058; PubMed Central PMCID: PMCPMC5082607. 10.12688/f1000research.9090.1 PMC5082607 27830058

[B18] Li B. Brady S. W. Ma X. Shen S. Zhang Y. Li Y. (2020). Therapy-induced Mutations Drive the Genomic Landscape of Relapsed Acute Lymphoblastic Leukemia. Blood 135 (1), 41–55. Epub 2019/11/08PubMed PMID: 31697823; PubMed Central PMCID: PMCPMC6940198. 10.1182/blood.2019002220 31697823 PMC6940198

[B19] Malim M. H. (2009). APOBEC Proteins and Intrinsic Resistance to HIV-1 Infection. Phil. Trans. R. Soc. B 364 (1517), 675–687. Epub 2008/11/29PubMed PMID: 19038776; PubMed Central PMCID: PMCPMC2660912. 10.1098/rstb.2008.0185 19038776 PMC2660912

[B20] Martincorena I. Campbell P. J. (2015). Somatic Mutation in Cancer and normal Cells. Science 349 (6255), 1483–1489. Epub 2015/09/26PubMed PMID: 26404825. 10.1126/science.aab4082 26404825

[B21] Mateo J. Carreira S. Sandhu S. Miranda S. Mossop H. Perez-Lopez R. (2015). DNA-repair Defects and Olaparib in Metastatic Prostate Cancer. N. Engl. J. Med. 373 (18), 1697–1708. Epub 2015/10/29PubMed PMID: 26510020; PubMed Central PMCID: PMCPMC5228595. 10.1056/NEJMoa1506859 26510020 PMC5228595

[B22] Merico D. Isserlin R. Stueker O. Emili A. Bader G. D. (2010). Enrichment Map: a Network-Based Method for Gene-Set Enrichment Visualization and Interpretation. PLoS One 5 (11), e13984. Epub 2010/11/19PubMed PMID: 21085593; PubMed Central PMCID: PMCPMC2981572. 10.1371/journal.pone.0013984 21085593 PMC2981572

[B23] Morganella S. Alexandrov L. B. Glodzik D. Zou X. Davies H. Staaf J. (2016). The Topography of Mutational Processes in Breast Cancer Genomes. Nat. Commun. 7, 11383. Epub 2016/05/03PubMed PMID: 27136393; PubMed Central PMCID: PMCPMC5001788. 10.1038/ncomms11383 27136393 PMC5001788

[B24] Negrini S. Gorgoulis V. G. Halazonetis T. D. (2010). Genomic Instability - an Evolving Hallmark of Cancer. Nat. Rev. Mol. Cel Biol 11 (3), 220–228. Epub 2010/02/24PubMed PMID: 20177397. 10.1038/nrm2858 20177397

[B25] Nik-Zainal S. Alexandrov L. B. Wedge D. C. Van Loo P. Greenman C. D. Raine K. (2012). Mutational Processes Molding the Genomes of 21 Breast Cancers. Cell 149 (5), 979–993. Epub 2012/05/23PubMed PMID: 22608084; PubMed Central PMCID: PMCPMC3414841. 10.1016/j.cell.2012.04.024 22608084 PMC3414841

[B26] Paczkowska M. Barenboim J. Barenboim J. Sintupisut N. Fox N. S. Zhu H. (2020). Integrative Pathway Enrichment Analysis of Multivariate Omics Data. Nat. Commun. 11 (1), 735. Epub 2020/02/07PubMed PMID: 32024846; PubMed Central PMCID: PMCPMC7002665. 10.1038/s41467-019-13983-9 32024846 PMC7002665

[B27] Peña-Diaz J. Bregenhorn S. Ghodgaonkar M. Follonier C. Artola-Borán M. Castor D. (2012). Noncanonical Mismatch Repair as a Source of Genomic Instability in Human Cells. Mol. Cel 47 (5), 669–680. Epub 2012/08/07PubMed PMID: 22864113. 10.1016/j.molcel.2012.07.006 22864113

[B28] Persi E. Wolf Y. I. Horn D. Ruppin E. Demichelis F. Gatenby R. A. (2021). Mutation-selection Balance and Compensatory Mechanisms in Tumour Evolution. Nat. Rev. Genet. 22 (4), 251–262. Epub 2020/12/02PubMed PMID: 33257848. 10.1038/s41576-020-00299-4 33257848

[B29] Pfeifer G. P. (2010). Environmental Exposures and Mutational Patterns of Cancer Genomes. Genome Med. 2 (8), 54. Epub 2010/08/17PubMed PMID: 20707934; PubMed Central PMCID: PMCPMC2945011. 10.1186/gm175 20707934 PMC2945011

[B30] Poulos R. C. Wong Y. T. Ryan R. Pang H. Wong J. W. H. (201810077). Analysis of 7,815 Cancer Exomes Reveals Associations between Mutational Processes and Somatic Driver Mutations. Plos Genet. 14 (11), e1007779. Epub 2018/11/10PubMed PMID: 30412573; PubMed Central PMCID: PMCPMC6249022. 10.1371/journal.pgen10.1371/journal.pgen.1007779 PMC6249022 30412573

[B31] Rayner E. van Gool I. C. Palles C. Kearsey S. E. Bosse T. Tomlinson I. (2016). A Panoply of Errors: Polymerase Proofreading Domain Mutations in Cancer. Nat. Rev. Cancer 16 (2), 71–81. Epub 2016/01/30PubMed PMID: 26822575. 10.1038/nrc.2015.12 26822575

[B32] Ripley W. N. V. B. D. (2002). Modern Applied Statistics with S. New York: Springer-Verlag.

[B33] Roberts S. A. Gordenin D. A. (2014). Hypermutation in Human Cancer Genomes: Footprints and Mechanisms. Nat. Rev. Cancer 14 (12), 786–800. Epub 2015/01/09PubMed PMID: 25568919; PubMed Central PMCID: PMCPMC4280484. 10.1038/nrc3816 25568919 PMC4280484

[B34] Roberts S. A. Lawrence M. S. Klimczak L. J. Grimm S. A. Fargo D. Stojanov P. (2013). An APOBEC Cytidine Deaminase Mutagenesis Pattern Is Widespread in Human Cancers. Nat. Genet. 45 (9), 970–976. Epub 2013/07/16PubMed PMID: 23852170; PubMed Central PMCID: PMCPMC3789062. 10.1038/ng.2702 23852170 PMC3789062

[B35] Sanchez-Vega F. Mina M. Armenia J. Chatila W. K. Luna A. La K. C. (2018). Oncogenic Signaling Pathways in the Cancer Genome Atlas. Cell 173 (2), 321–e10. Epub 2018/04/07PubMed PMID: 29625050; PubMed Central PMCID: PMCPMC6070353. 10.1016/j.cell.2018.03.035 29625050 PMC6070353

[B36] Shannon P. Markiel A. Ozier O. Baliga N. S. Wang J. T. Ramage D. (2003). Cytoscape: a Software Environment for Integrated Models of Biomolecular Interaction Networks. Genome Res. 13 (11), 2498–2504. Epub 2003/11/05PubMed PMID: 14597658; PubMed Central PMCID: PMCPMC403769. 10.1101/gr.1239303 14597658 PMC403769

[B37] Shi M.-J. Meng X.-Y. Lamy P. Banday A. R. Yang J. Moreno-Vega A. (2019). APOBEC-mediated Mutagenesis as a Likely Cause of FGFR3 S249C Mutation Over-representation in Bladder Cancer. Eur. Urol. 76 (1), 9–13. Epub 2019/04/13PubMed PMID: 30975452. 10.1016/j.eururo.2019.03.032 30975452

[B38] Stavrou S. Ross S. R. (2015). APOBEC3 Proteins in Viral Immunity. J.I. 195 (10), 4565–4570. Epub 2015/11/08PubMed PMID: 26546688; PubMed Central PMCID: PMCPMC4638160. 10.4049/jimmunol.1501504 PMC4638160 26546688

[B39] Telli M. L. Timms K. M. Reid J. Hennessy B. Mills G. B. Jensen K. C. (2016). Homologous Recombination Deficiency (HRD) Score Predicts Response to Platinum-Containing Neoadjuvant Chemotherapy in Patients with Triple-Negative Breast Cancer. Clin. Cancer Res. 22 (15), 3764–3773. Epub 2016/03/10PubMed PMID: 26957554; PubMed Central PMCID: PMCPMC6773427. 10.1158/1078-0432.CCR-15-2477 26957554 PMC6773427

[B40] Temko D. Tomlinson I. P. M. Severini S. Schuster-Böckler B. Graham T. A. (2018). The Effects of Mutational Processes and Selection on Driver Mutations across Cancer Types. Nat. Commun. 9 (1), 1857. Epub 2018/05/12PubMed PMID: 29748584; PubMed Central PMCID: PMCPMC5945620. 10.1038/s41467-018-04208-6 29748584 PMC5945620

[B41] Tokheim C. J. Papadopoulos N. Kinzler K. W. Vogelstein B. Karchin R. (2016). Evaluating the Evaluation of Cancer Driver Genes. Proc. Natl. Acad. Sci. USA 113 (50), 14330–14335. Epub 2016/12/03PubMed PMID: 27911828; PubMed Central PMCID: PMCPMC5167163. 10.1073/pnas.1616440113 27911828 PMC5167163

[B42] Trusolino L. Bertotti A. Comoglio P. M. (2010). MET Signalling: Principles and Functions in Development, Organ Regeneration and Cancer. Nat. Rev. Mol. Cel Biol 11 (12), 834–848. Epub 2010/11/26PubMed PMID: 21102609. 10.1038/nrm3012 21102609

[B43] Van Allen E. M. Mouw K. W. Kim P. Iyer G. Wagle N. Al-Ahmadie H. (2014). Somatic ERCC2 Mutations Correlate with Cisplatin Sensitivity in Muscle-Invasive Urothelial Carcinoma. Cancer Discov. 4 (10), 1140–1153. Epub 2014/08/07PubMed PMID: 25096233; PubMed Central PMCID: PMCPMC4238969. 10.1158/2159-8290.CD-14-0623 25096233 PMC4238969

[B44] Vieira V. C. Soares M. A. (2013). The Role of Cytidine Deaminases on Innate Immune Responses against Human Viral Infections. Biomed. Res. Int. 2013, 1–18. Epub 2013/07/19PubMed PMID: 23865062; PubMed Central PMCID: PMCPMC3707226. 10.1155/2013/683095 PMC3707226 23865062

[B45] Walker C. Mojares E. del Río Hernández A. (2018). Role of Extracellular Matrix in Development and Cancer Progression. Ijms 19 (10), 3028. Epub 2018/10/06PubMed PMID: 30287763; PubMed Central PMCID: PMCPMC6213383. 10.3390/ijms19103028 PMC6213383 30287763

[B46] Wang F. Zhao Q. Wang Y.-N. Jin Y. He M.-M. Liu Z.-X. (2019). Evaluation of POLE and POLD1 Mutations as Biomarkers for Immunotherapy Outcomes across Multiple Cancer Types. JAMA Oncol. 5, 1504. Epub 2019/08/16PubMed PMID: 31415061; PubMed Central PMCID: PMCPMC6696731. 10.1001/jamaoncol.2019.2963 31415061 PMC6696731

[B47] Wei R. Li P. He F. Wei G. Zhou Z. Su Z. (2020). Comprehensive Analysis Reveals Distinct Mutational Signature and its Mechanistic Insights of Alcohol Consumption in Human Cancers. Brief Bioinform 22. Epub 2020/06/02PubMed PMID: 32480415. 10.1093/bib/bbaa066 32480415

