# Supplementary material for: Analysis of Mutations and Dysregulated Pathways Unravels Carcinogenic Effect and Clinical Actionability of Mutational Processes
Source: Front Cell Dev Biol. 2021 Nov 24;9:768981. doi: 10.3389/fcell.2021.768981 (PMC8652146; doi:10.3389/fcell.2021.768981)
Supplement: Supplementary file 4 [file DataSheet1.pdf]

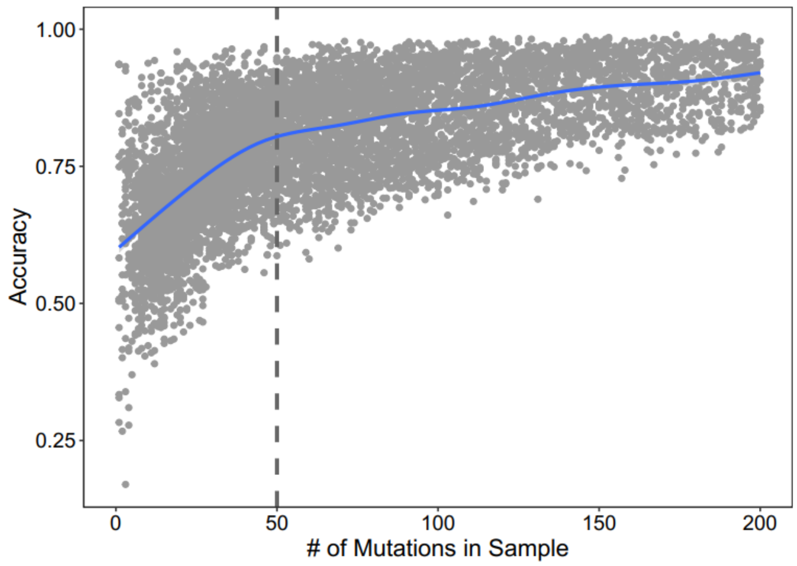


**Fig. S1. The relationship between sample mutation number and mutation signature fitting accuracy.** The x-axes and y-axes show the mutation number in samples and mutational signature fitting accuracy respectively.

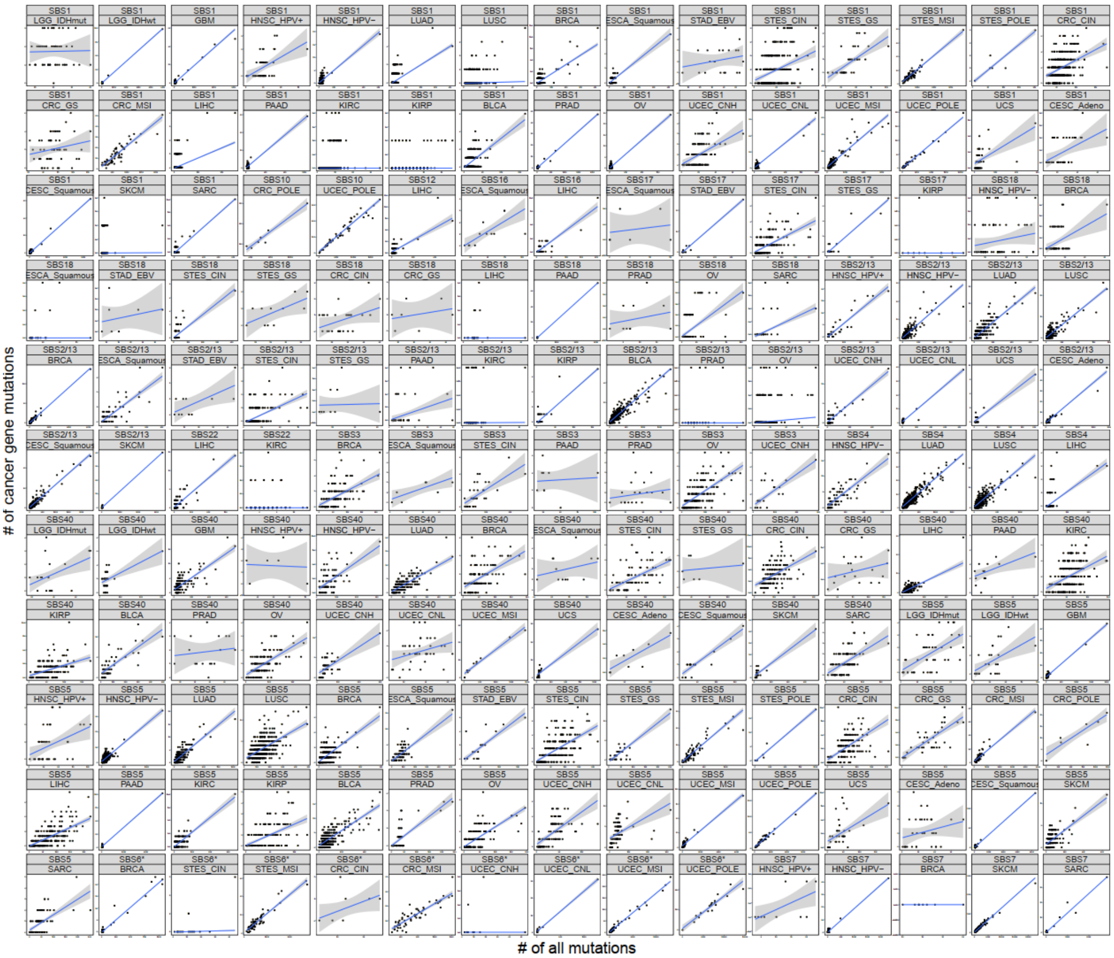


**Fig. S2. Correlations between the number of cancer gene mutations and total mutations caused by mutational processes.** The y-axes and x-axes show the numbers of cancer gene mutations and total mutations attributed to each mutational process respectively. Each panel corresponds to a mutational process in a cancer type or subtype. Each dot represents a sample. The lines represent the linear relationship calculated by robust linear regression and 95% confidence intervals for the slopes are shown in lighter gray shading.

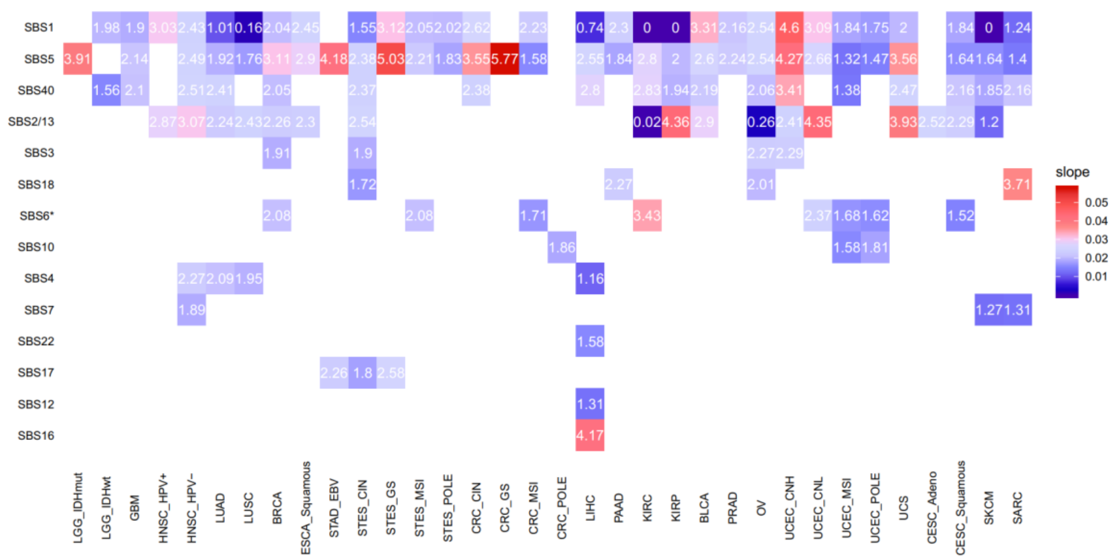

**Fig. S3. The cancer gene mutation risk of mutational processes in each cancer type.** Detailed heatmap of significant cancer gene mutation risk of mutational processes in each cancer type (see Methods). The filled color of the heatmap represents the degree of risk. The risk value on the heat map is multiplied by 100 for the sake of showing it.

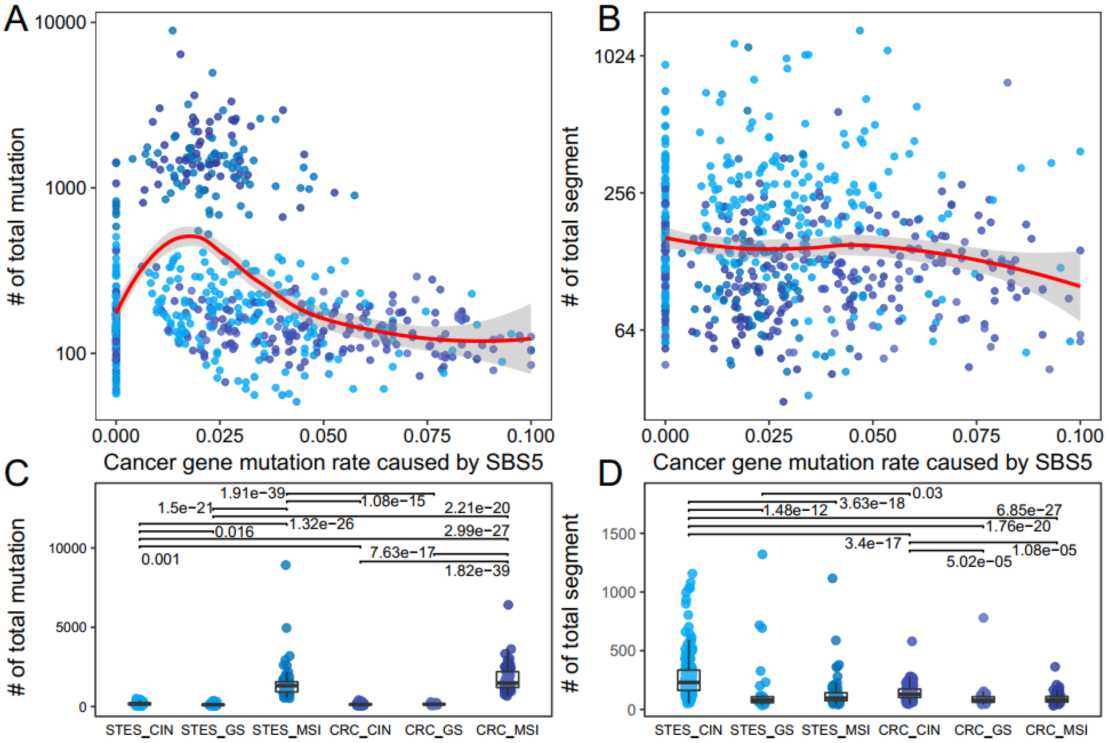


**Fig. S4. The relationship between cancer gene mutation risk induced by SBS5 and genomic instability.** (A) The relationship between cancer gene mutation risk induced by SBS5 and mutation burden. (B) The relationship between cancer gene mutation risk induced by SBS5 and copy number burden. (C) Mutation load differences among GIACs subtypes. (D) Copy number load differences among GIACs subtypes.

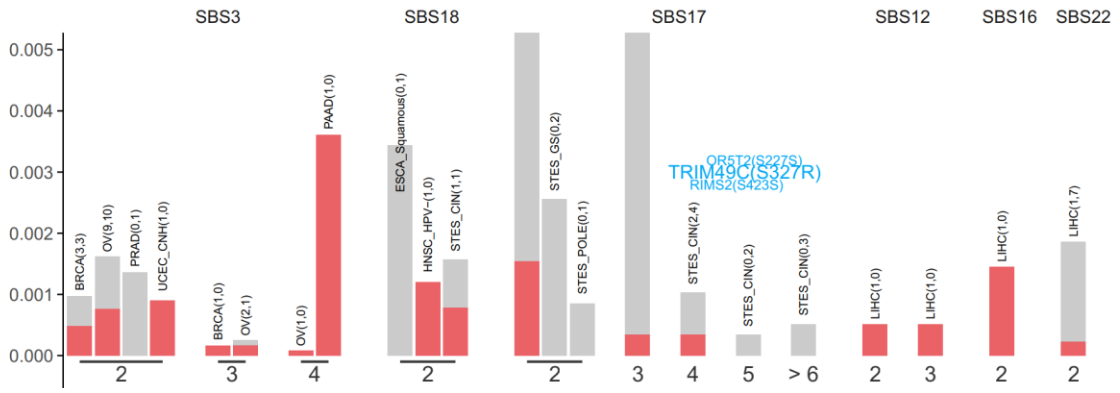


**Fig. S5. The recurrent mutation is shaped by some mutational processes.** Barplots and word clouds illustrate the recurrent mutation landscapes for SBS3, SBS18, SBS17, SBS12, SBS16, SBS22. Barplot depicts a recurrence pattern, where mutations were binned by their reoccurrence frequency and the height of the bar represented the fraction of mutations in each cancer type or subtype. The numbers of mutations in and out of cancer genes are filled by red and gray respectively and also show in parentheses separated by commas in each cancer type or subtype. Word clouds show high-frequency mutations occurring at least 6 times in a cancer type or subtype, of which word size is proportional to the number of mutations and word colored by cancer type or subtype. Note that some mutational processes are not found to cause high-frequency mutations.

| | SBS1 | SBS5 | SBS40 | SBS2/13 | SBS3 | SBS18 | SBS6* | SBS10 | SBS4 | SBS7 | SBS22 | SBS17 | SBS12 | SBS16 |
|---|---|---|---|---|---|---|---|---|---|---|---|---|---|---|
| SARC | 127 | 78 | 92 | 3 | | 22 | 1 | | | 62 | | 1 | | |
| SKCM | 283 | 337 | 25 | 26 | | | | | | 365 | | 1 | 1 | |
| CESC_Squamous | 205 | 193 | 18 | 197 | | 3 | 4 | | | | | | | |
| CESC_Adeno | 38 | 33 | 9 | 35 | | 1 | | | | | | 1 | | |
| UCS | 46 | 31 | 23 | 19 | | | 1 | 1 | 3 | | | | | |
| UCEC_POLE | 40 | 42 | 5 | 1 | | | 18 | 43 | | | | | | |
| UCEC_MSI | 135 | 135 | 12 | 1 | 5 | | 140 | 4 | | | | | | |
| UCEC_CNL | 111 | 92 | 39 | 29 | 3 | | 12 | | | | | | | |
| UCEC_CNH | 118 | 94 | 44 | 76 | 20 | | 10 | | | 1 | | | | |
| OV | 293 | 169 | 118 | 199 | 189 | 75 | 1 | | | 3 | 1 | | | |
| PRAD | 66 | 49 | 17 | 28 | 20 | 10 | 1 | | | 1 | | | | |
| BLCA | 343 | 349 | 53 | 359 | | 3 | 1 | 1 | | 1 | 1 | | 1 | |
| KIRP | 115 | 144 | 144 | 43 | | | 2 | | | | 5 | 9 | 2 | |
| KIRC | 201 | 119 | 219 | 66 | | | 4 | | 1 | 2 | 61 | 4 | | |
| PAAD | 74 | 59 | 13 | 30 | 9 | 7 | 1 | | | 1 | | | | |
| LIHC | 212 | 221 | 158 | 2 | | 22 | 2 | | 63 | 2 | 133 | 2 | 73 | 40 |
| CRC_POLE | 3 | 7 | | | | | 4 | 7 | | | | | | |
| CRC_MSI | 59 | 61 | | | | | 59 | | | | | | | |
| CRC_GS | 53 | 38 | 20 | 1 | | 7 | 2 | | | 1 | | 1 | | |
| CRC_CIN | 299 | 185 | 156 | 2 | | 25 | 7 | | 1 | | | 3 | | |
| STES_POLE | 6 | 6 | 1 | 3 | 3 | | 3 | 1 | | | | 4 | | |
| STES_MSI | 69 | 72 | | | 3 | 1 | 70 | | | | | 4 | | |
| STES_GS | 34 | 30 | 7 | 9 | 3 | 11 | 2 | | | | | 20 | | |
| STES_CIN | 273 | 221 | 74 | 136 | 34 | 60 | 17 | | | | 1 | 171 | | |
| STAD_EBV | 21 | 17 | 5 | 11 | 3 | 7 | | | | | | 10 | | |
| ESCA_Squamous | 82 | 80 | 12 | 69 | 8 | 19 | | | 2 | | | 6 | | 12 |
| BRCA | 468 | 338 | 109 | 409 | 121 | 47 | 41 | 1 | | 8 | | 1 | | |
| LUSC | 242 | 413 | | 350 | | 1 | 4 | | 370 | 2 | | | | |
| LUAD | 346 | 255 | 200 | 253 | | | 1 | | 358 | 3 | | 1 | | |
| HNSC_HPV- | 346 | 323 | 81 | 296 | | 59 | 3 | | 72 | 131 | | | | 3 |
| HNSC_HPV+ | 41 | 36 | 6 | 37 | | 3 | | | 1 | 10 | | | | |
| GBM | 292 | 114 | 208 | 5 | | | | 2 | | 1 | | | | |
| LGG_IDHwt | 50 | 24 | 33 | | | | 1 | | | | | | | |
| LGG_IDHmut | 58 | 44 | 19 | 5 | | | 1 | | | | | | | |

**Fig. S6. The sample size affected by each mutation process in each cancer type.** The red numbers indicate that the mutational process causes high-frequency recurrent mutations in a cancer type.

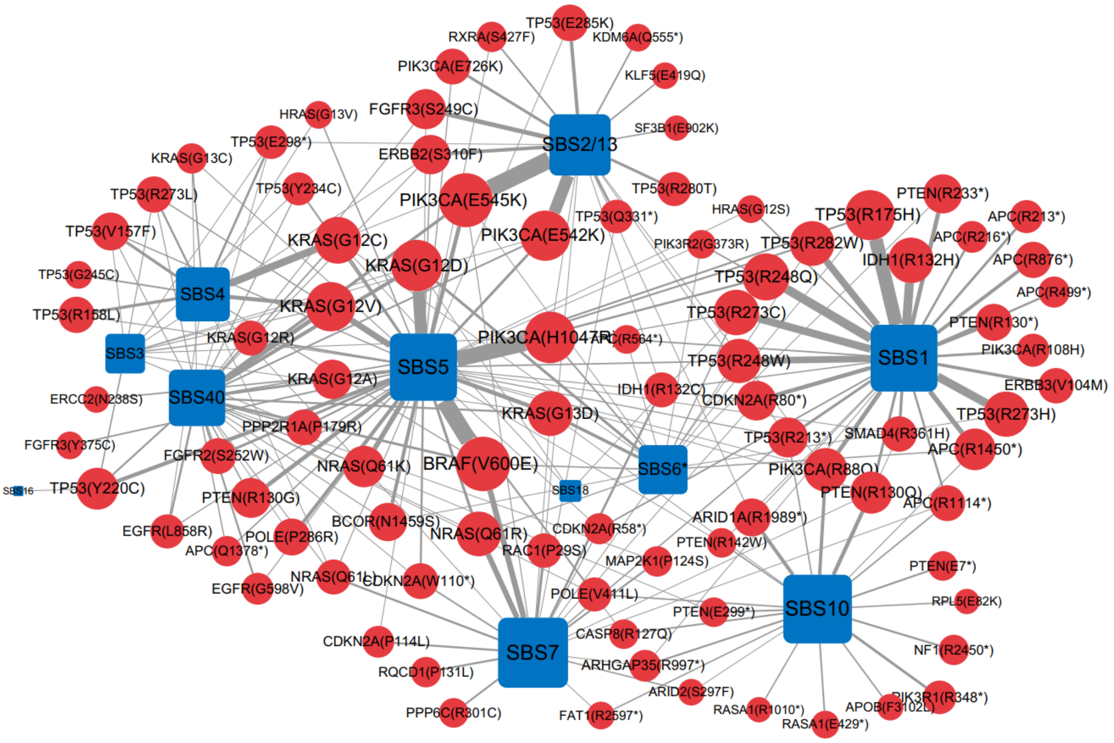


**Fig. S7. The relation between mutational processes and high-frequency mutations.** The network illustrates the associations between mutational processes and high-frequency mutations. Red circles and blue rectangles correspond to mutations and mutational processes, respectively. The size of the node is proportional to the degree in the network. The thickness of the edge connecting two nodes is proportional to the number of associations in all samples.

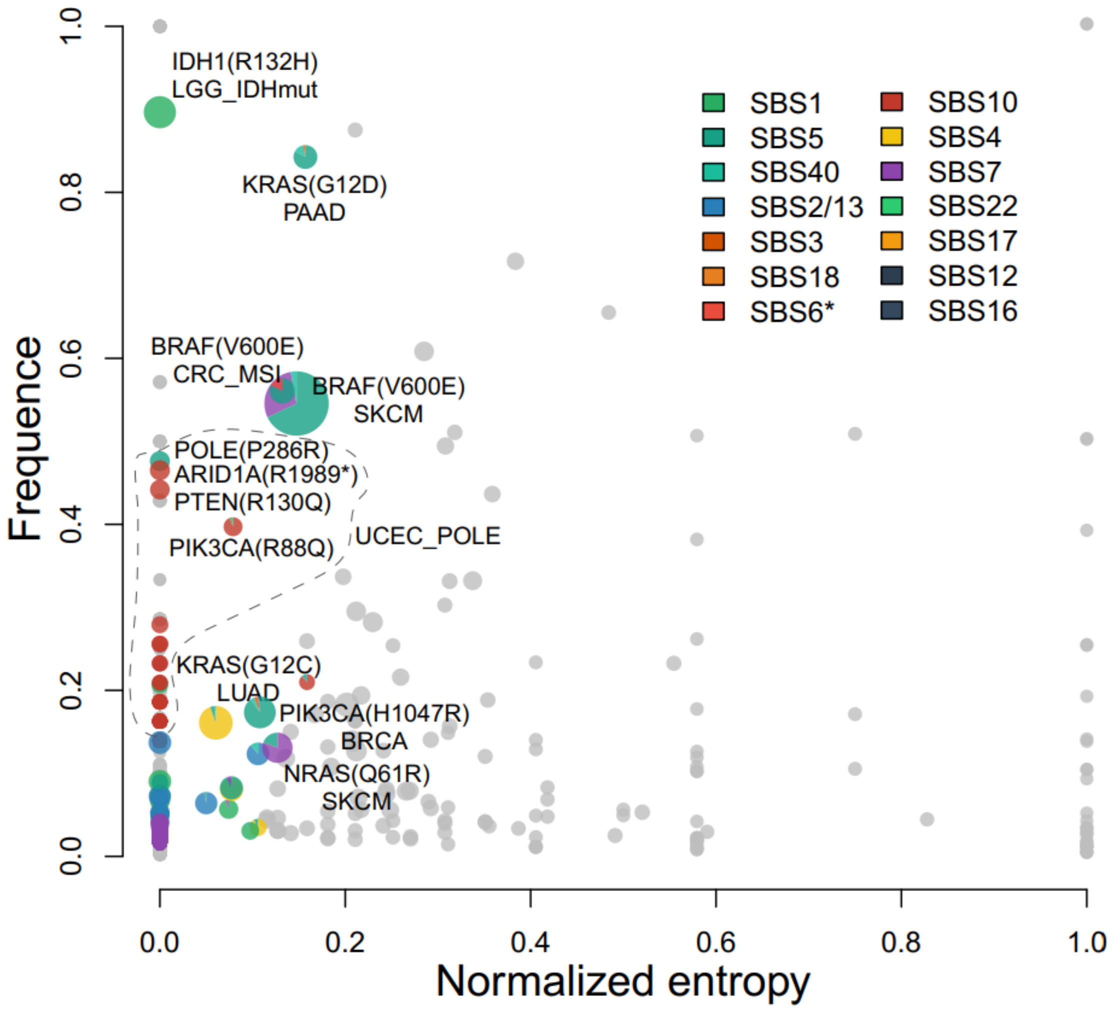


**Fig. S8. Specific mutations induced by a certain mutational process.** Pie charts show the mutational process composition contributing to a mutation in a cancer type and the size is proportional to the mutation number. Significant dots (adjusted $p < 0.05$) are filled by the mutational process. The y coordinate of each pie center reflects mutation frequency in the corresponding cancer type; the x coordinate was determined by the normalized entropy (see Methods).

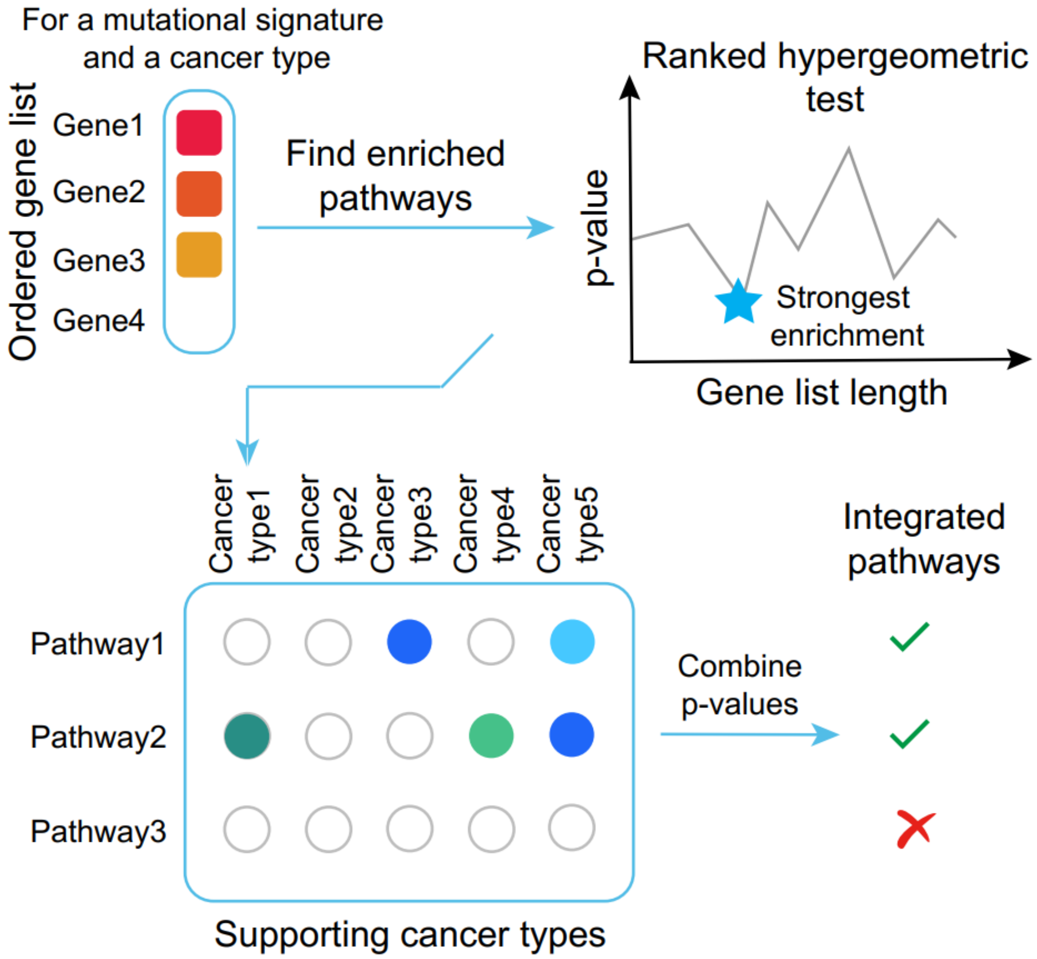


**Fig. S9. Overview of identifying pathways affected by the mutational process.** For a mutational process, firstly, we prioritized genes in a cancer type. Then, a ranked hypergeometric test is used to find enriched pathways. Finally, we get pathways by integrating the evidence from all cancer types. This schematic map is referenced by Paczkowska et al (Nature communication. 2020).

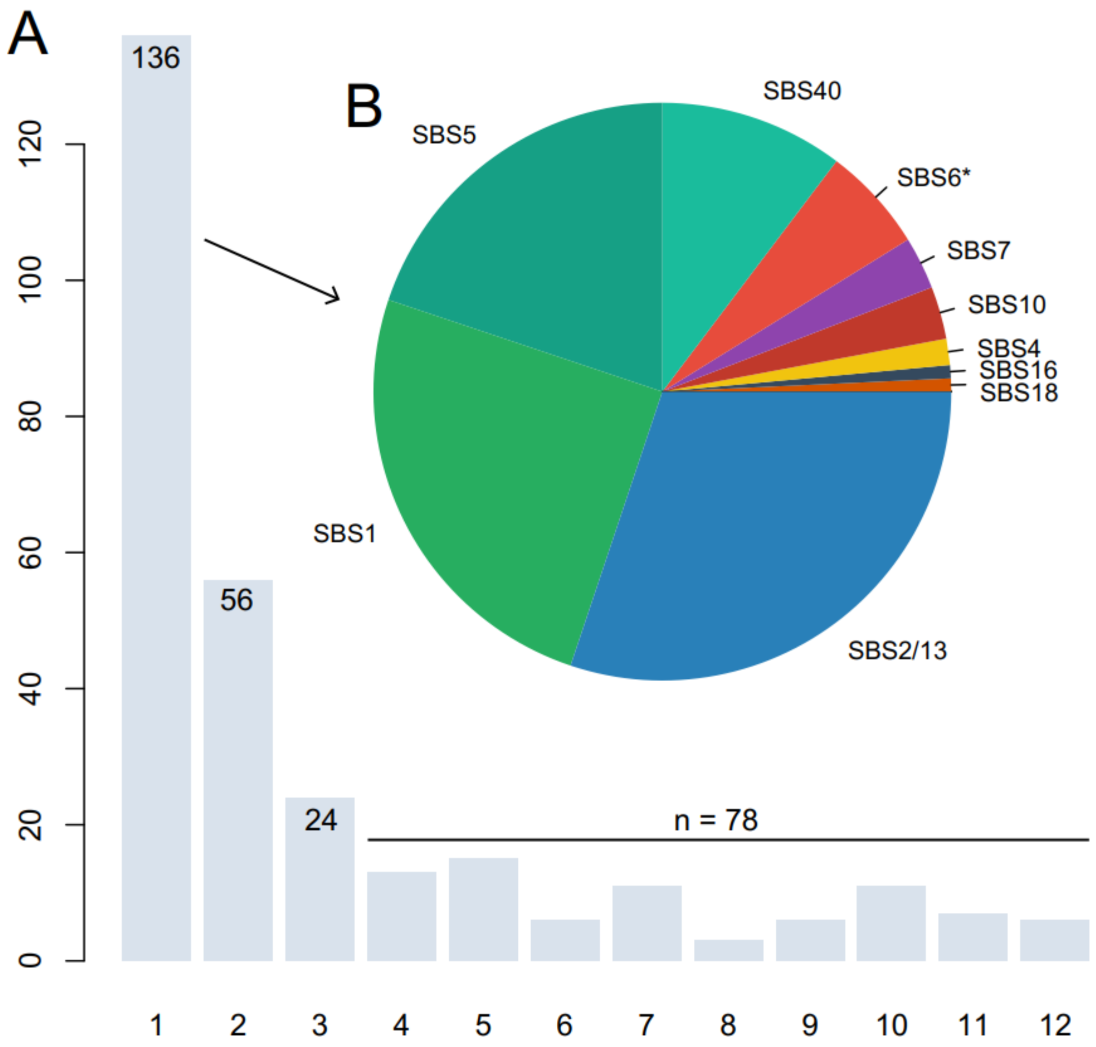


**Fig. S10. Summary of pathways affected by mutational processes.** (A) Barplot indicates the number of pathways (y-axis) affected by a certain number of the mutational process(es) (x-axis). (B) The pie chart shows the detailed composition of related mutational processes of the pathways that are specifically affected by a single mutational process.

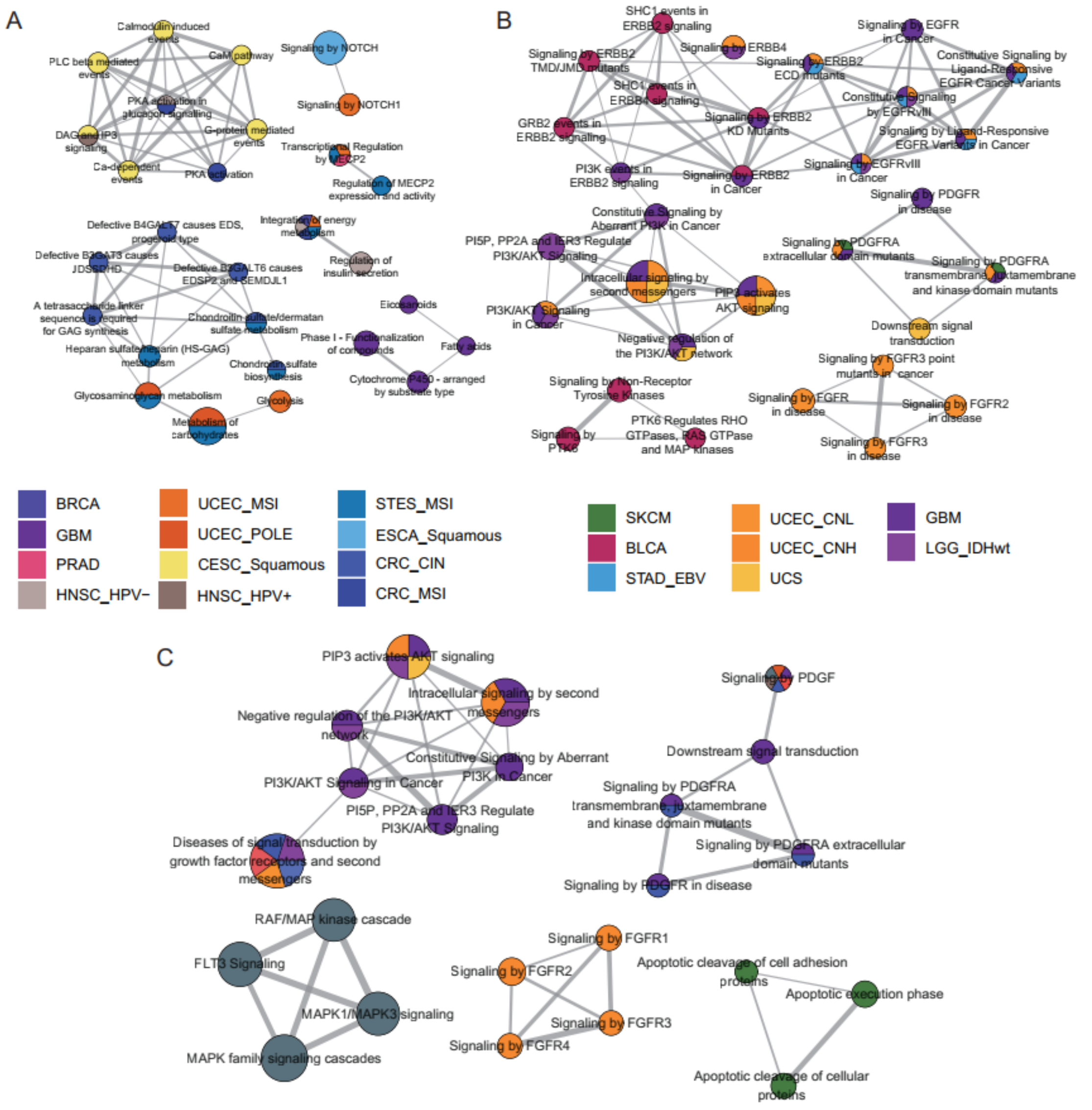


**Fig. S11. Pathways affected by clock-like mutational processes.** Enrichment map of some pathways affected by **A** SBS1, **B** SBS5, and **C** SBS40. Nodes in the network represent pathways and are filled by cancer-type evidence. The node size indicated the number of genes in a pathway. Similar pathways with many common genes were connected.

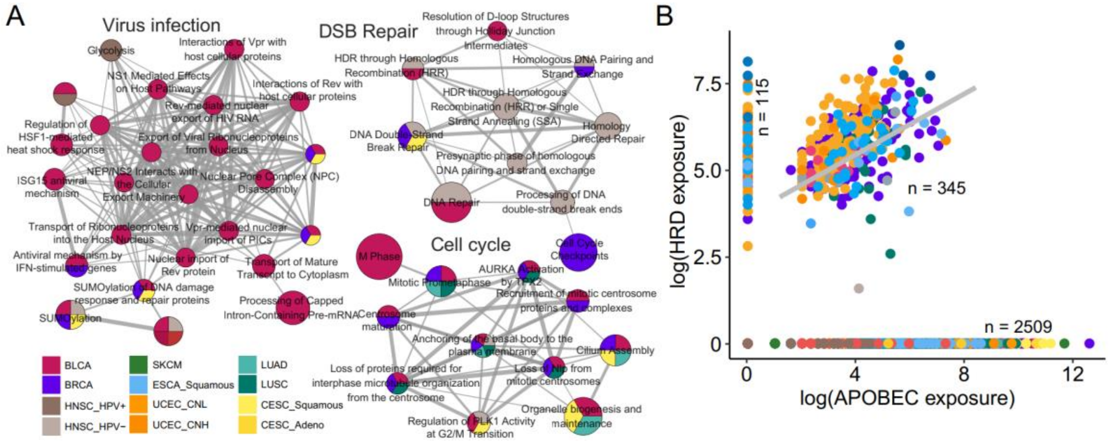


**Fig. S12. Pathways affected by APOBEC mutational process. (A)** Enrichment map of some pathways affected by APOBEC mutational process solely. In this map, nodes in the network represent pathways and are colored by cancer-type evidence. The node size indicates the number of genes in a pathway. Similar pathways with many common genes were connected. **(B)** A scatter plot shows correlations between the log exposure value of APOBEC mutational signatures (SBS2 and SBS13, axes) and HRD signature (SBS3, y-axes). Each dot represents a sample colored according to the cancer type and the line shows best estimates for the slope estimated by mixed effect model in samples that APOBEC mutational signatures and HRD signature co-occurred. The 0 value of exposure is not processed by log transformation.

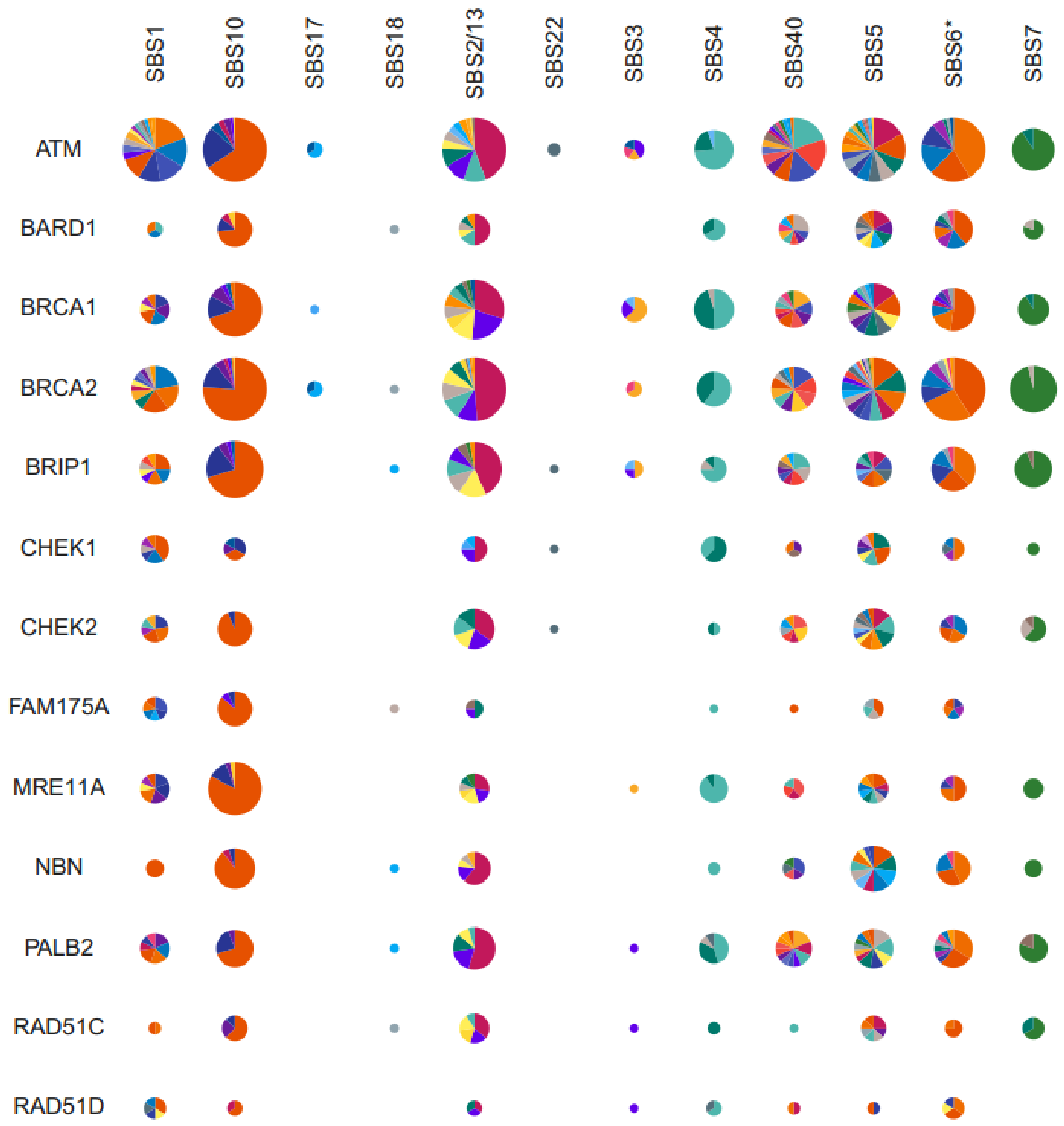


**Fig. S13. HR-related genes affected by various mutational processes.** The size of the pie chart represents the number of mutations caused by the mutational process in all cancer types, the color represents the different cancer types, and the area is proportional to the number of mutations in the corresponding cancer subtype.

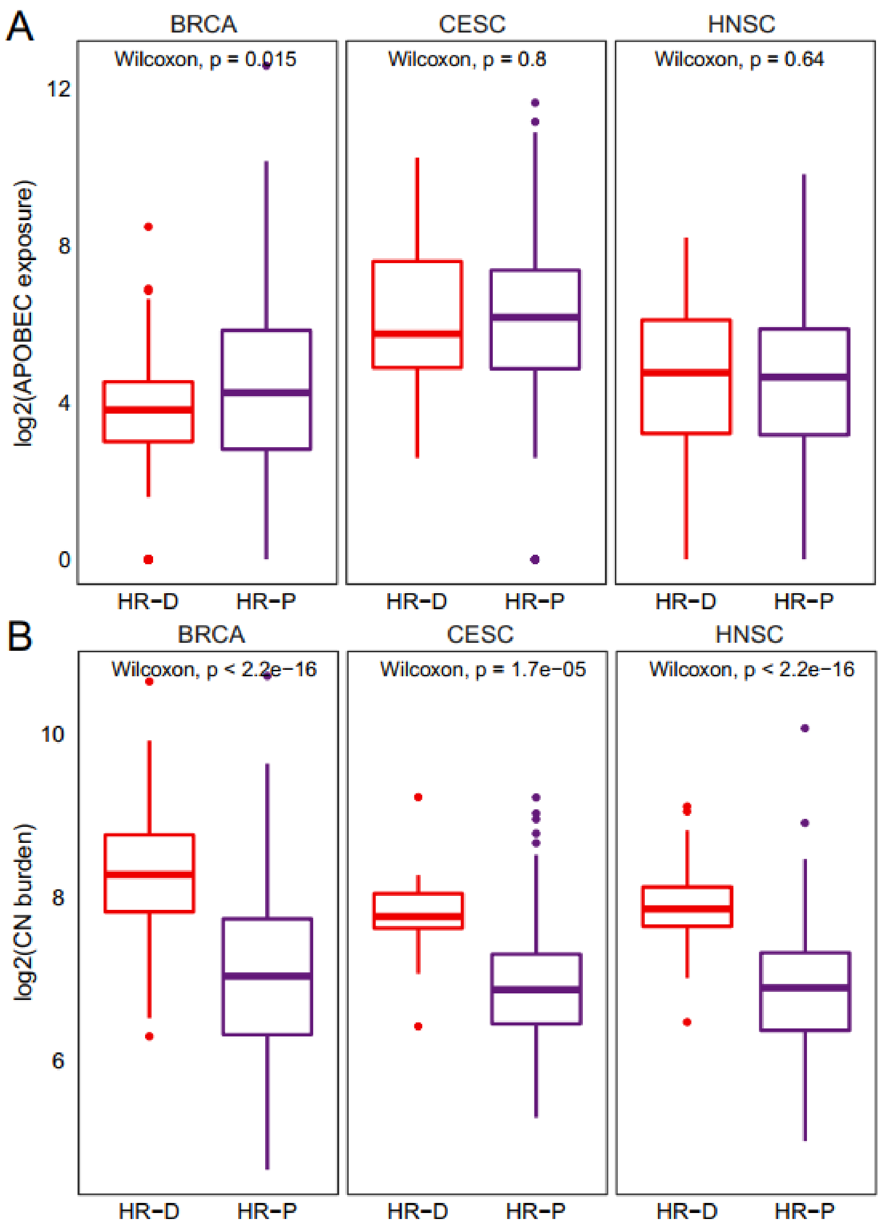


**Fig. S14. Differences between HR-Deficient and HR-Proficient groups in BRCA, CESC, and HNSC.** (A) Differences in APOBEC exposure between HRD and HRP groups. (B) Differences in copy number burden between HRD and HRP groups.

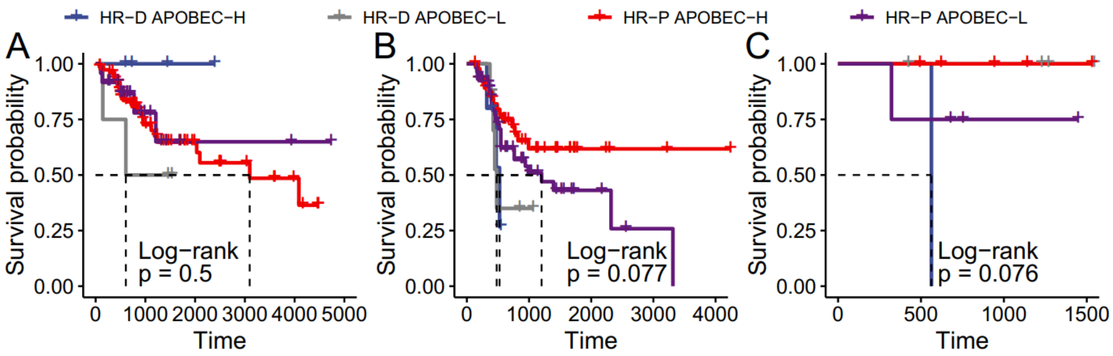


**Fig. S15. The contribution of the APOBEC mutation process to platinum-based therapy.** Kaplan–Meier curves for different HR status and APOBEC exposure levels in (A) CESC, (B) HNSC, and (C) BRCA patients.
